# *POGLUT1*, the putative effector gene driven by rs2293370 in primary biliary cholangitis susceptibility locus chromosome 3q13.33

**DOI:** 10.1038/s41598-018-36490-1

**Published:** 2019-01-14

**Authors:** Yuki Hitomi, Kazuko Ueno, Yosuke Kawai, Nao Nishida, Kaname Kojima, Minae Kawashima, Yoshihiro Aiba, Hitomi Nakamura, Hiroshi Kouno, Hirotaka Kouno, Hajime Ohta, Kazuhiro Sugi, Toshiki Nikami, Tsutomu Yamashita, Shinji Katsushima, Toshiki Komeda, Keisuke Ario, Atsushi Naganuma, Masaaki Shimada, Noboru Hirashima, Kaname Yoshizawa, Fujio Makita, Kiyoshi Furuta, Masahiro Kikuchi, Noriaki Naeshiro, Hironao Takahashi, Yutaka Mano, Haruhiro Yamashita, Kouki Matsushita, Seiji Tsunematsu, Iwao Yabuuchi, Hideo Nishimura, Yusuke Shimada, Kazuhiko Yamauchi, Tatsuji Komatsu, Rie Sugimoto, Hironori Sakai, Eiji Mita, Masaharu Koda, Yoko Nakamura, Hiroshi Kamitsukasa, Takeaki Sato, Makoto Nakamuta, Naohiko Masaki, Hajime Takikawa, Atsushi Tanaka, Hiromasa Ohira, Mikio Zeniya, Masanori Abe, Shuichi Kaneko, Masao Honda, Kuniaki Arai, Teruko Arinaga-Hino, Etsuko Hashimoto, Makiko Taniai, Takeji Umemura, Satoru Joshita, Kazuhiko Nakao, Tatsuki Ichikawa, Hidetaka Shibata, Akinobu Takaki, Satoshi Yamagiwa, Masataka Seike, Shotaro Sakisaka, Yasuaki Takeyama, Masaru Harada, Michio Senju, Osamu Yokosuka, Tatsuo Kanda, Yoshiyuki Ueno, Hirotoshi Ebinuma, Takashi Himoto, Kazumoto Murata, Shinji Shimoda, Shinya Nagaoka, Seigo Abiru, Atsumasa Komori, Kiyoshi Migita, Masahiro Ito, Hiroshi Yatsuhashi, Yoshihiko Maehara, Shinji Uemoto, Norihiro Kokudo, Masao Nagasaki, Katsushi Tokunaga, Minoru Nakamura

**Affiliations:** 1 Department of Human Genetics, Graduate School of Medicine, the University of Tokyo, Tokyo, Japan; 2 Department of Integrative Genomics, Tohoku Medical Megabank Organization, Tohoku University, Sendai, Japan; 3 Graduate School of Medicine, Tohoku University, Sendai, Japan; 4 The Research Center for Hepatitis and Immunology, National Center for Global Health and Medicine, Ichikawa, Japan; 5 Japan Science and Technology Agency (JST), Tokyo, Japan; 6 Clinical Research Center, National Hospital Organization (NHO) Nagasaki Medical Center, Omura, Japan; 7 Headquarters of PBC Research in NHO Study Group for Liver Disease in Japan (NHOSLJ), Clinical Research Center, NHO Nagasaki Medical Center, Omura, Japan; 8 Department of Medicine, Teikyo University School of Medicine, Tokyo, Japan; 9 Department of Gastroenterology and Rheumatic Diseases, Fukushima Medical University of Medicine, Fukushima, Japan; 10 Department of Gastroenterology and Hepatology, Tokyo Jikei University School of Medicine, Tokyo, Japan; 11 Department of Gastroenterology and Metabology, Ehime University Graduate School of Medicine, Matsuyama, Japan; 12 Department of Gastroenterology, Kanazawa University Graduate School of Medicine, Kanazawa, Japan; 13 Division of Gastroenterology, Department of Medicine, Kurume University School of Medicine, Kurume, Japan; 14 Department of Medicine and Gastroenterology, Tokyo Women’s Medical University, Tokyo, Japan; 15 Department of Medicine, Division of Gastroenterology and Hepatology, Shinshu University School of Medicine, Matsumoto, Japan; 16 Department of Gastroenterology and Hepatology, Nagasaki University Graduate School of Biomedical Sciences, Nagasaki, Japan; 17 Department of Gastroenterology and Hepatology, Okayama University Graduate School of Medicine, Dentistry and Pharmaceutical Sciences, Okayama, Japan; 18 Division of Gastroenterology and Hepatology, Niigata University Graduate School of Medical and Dental Sciences, Niigata, Japan; 19 Faculty of Medicine, Oita University, Oita, Japan; 20 Department of Gastroenterology and Medicine, Fukuoka University School of Medicine, Fukuoka, Japan; 21 The Third Department of Internal Medicine, School of Medicine, University of Occupational and Environmental Health, Kitakyushu, Japan; 22 Department of Medicine and Clinical Oncology, Graduate School of Medicine, Chiba University, Chiba, Japan; 23 Department of Gastroenterology, Yamagata University Faculty of Medicine, Yamagata, Japan; 24 Division of Gastroenterology and Hepatology, Department of Internal Medicine, Keio Graduate School of Medicine, Tokyo, Japan; 25 Department of Medical Technology, Kagawa Prefectural University of Health Sciences, Kagawa, Japan; 26 Department of Medicine and Biosystemic Science, Kyushu University Graduate School of Medical Sciences, Fukuoka, Japan; 27 Department of Hepatology, Nagasaki University Graduate School of Biomedical Sciences, Omura, Japan; 28 Department of Surgery and Science, Kyushu University Graduate School of Medical Sciences, Fukuoka, Japan; 29 Division of Hepato-Biliary-Pancreatic and Transplant Surgery, Department of Surgery, Graduate School of Medicine, Kyoto University, Kyoto, Japan; 30 National Center for Global Health and Medicine, Tokyo, Japan; 31 Graduate School of Information Sciences, Tohoku University, Sendai, Japan; 32 Headquaters of PBC-GWAS study group in Japan, Clinical Research Center, NHO Nagasaki Medical Center, Omura, Japan

## Abstract

Primary biliary cholangitis (PBC) is a chronic and cholestatic autoimmune liver disease caused by the destruction of intrahepatic small bile ducts. Our previous genome-wide association study (GWAS) identified six susceptibility loci for PBC. Here, in order to further elucidate the genetic architecture of PBC, a GWAS was performed on an additional independent sample set, then a genome-wide meta-analysis with our previous GWAS was performed based on a whole-genome single nucleotide polymorphism (SNP) imputation analysis of a total of 4,045 Japanese individuals (2,060 cases and 1,985 healthy controls). A susceptibility locus on chromosome 3q13.33 (including *ARHGAP*3*1*, *TMEM39A*, *POGLUT1*, *TIMMDC1*, and *CD80*) was previously identified both in the European and Chinese populations and was replicated in the Japanese population (OR = 0.7241, *P* = 3.5 × 10^−9^). Subsequent *in silico* and *in vitro* functional analyses identified rs2293370, previously reported as the top-hit SNP in this locus in the European population, as the primary functional SNP. Moreover, e-QTL analysis indicated that the effector gene of rs2293370 was *Protein O-Glucosyltransferase 1* (*POGLUT1*) (*P* = 3.4 × 10^−8^). This is the first study to demonstrate that *POGLUT1* and not *CD80* is the effector gene regulated by the primary functional SNP rs2293370, and that increased expression of *POGLUT1* might be involved in the pathogenesis of PBC.

## Introduction

Primary biliary cholangitis (PBC) is a chronic and progressive cholestatic liver disease characterized by chronic non-suppurative destructive cholangitis (CNSDC), ductopenia, interface hepatitis, fibrosis, and biliary cirrhosis^1,2^. The destruction of small bile ducts is considered to be mediated by autoimmune responses against biliary epithelial cells (BEC), including CD4^+^ T cells, CD8^+^ T cells, B cells, and natural killer (NK) cells^2–4^. The higher monozygotic/dizygotic (MZ/DZ) ratio and the higher estimated relative sibling risk (λs) in PBC patients as compared to unaffected individuals indicates the involvement of strong genetic factors in the development of PBC^5,6^. Previous genome-wide association studies (GWASs), ImmunoChip analyses, and subsequent meta-analyses in populations of European descent identified human leukocyte antigen (*HLA*) and 30 non-*HLA* susceptibility regions (nearest candidate genes from the top-hit SNPs in each locus: *IL12RB2*/*DENND1B*/*C11orf5*3, *YPEL5*/*LBH*, *IL1RL1*/*IL1RL2*, *STAT4*/*NAB1*, *SLC19A3*/*CCL20*, *PLCL2*, *TIMMDC1*/*TMEM39A*, *IL-12A*/*IL12A-AS1*/*IQCJ*/*SCHIP1*, *DGKQ*, *NFKB1*/*MANBA*, *IL7R*/*CAPSL*, *NUDT12*/*C5orf30*, *IL12B*, *OLIG3*/*TNFAIP3*, *ELMO1*, *IRF5*/*TNPO3*, *RPS6KA4*, *DDX6*/*CXCR5*, *TNFRSF1A*, *ATXN2*/*BRAP*, *DLEU1*/*BCMS*, *RAD51B*, *EXOC3L4*, *RMI2*/*CLEC16A*, *IRF8*/*FOXF1*, *ZPBP2*/*GSDMB*/*IKZF3*, *MAPT*, *TYK2*, *SPIB*, and *SYNGR1*/*PDGFB*/*RPL3*) in PBC^7–14^. Additionally, Asian-specific susceptibility regions for PBC, including *CD58*, *CD28*/*CTLA4*, *IL21-AS1*, *TNFSF15*/*TNFSF8*, *IL16*, *IL21R*, *CSNK2N2*/*CCDC113*, and *AATID3A*, were reported in the Japanese and Chinese populations by means of GWAS and subsequent genome-wide meta-analysis with genome-wide SNP imputation (already identified PBC susceptibility loci including this study are shown in Table 1)^15–17^. Thus, the evidence reported to date indicates presence of shared and non-shared genetic susceptibility profiles behind the pathogenesis of PBC in European and Asian populations.

**Table 1:** Non-*HLA* PBC susceptibility loci in European, Chinese, and Japanese populations.

Chromosome	Location	Mapped gene(s)	Top-hit SNP	MAF^a^	OR^b^	*P*	Population	Ref.
1	1p13.1	*CD58*	rs2300747	0.39	1.29	2.E-12	Chinese	^17^
	1p31.3	*IL12RB2*/*DENND1B*/*C1orf53*	rs72678531	0.16	1.61	2.E-38	European	^12^
2	2p23.1	*YPEL5*/*LBH*	rs4952108	0.19	1.28	5.E-08	European	^14^
	2q12.1	*IL1RL1*/*IL1RL2*	rs12712133	0.44	1.14	5.E-09	European	^14^
	2q32.2	*STAT4*/*NAB1*	rs3024921, etc.	0.05	0.72	9.E-25	European	^12^
			rs10168266	0.32	1.31	4.E-14	Chinese	^17^
	2q33.2	*CD28*/*CTLA4*	rs4675369	0.42	1.31	1.E-13	Chinese	^17^
	2q36.3	*SLC19A3*/*CCL20*	rs4973341	0.33	1.22	2.E-10	European	^14^
3	3p24.3	*PLCL2*	rs1372072	0.37	1.20	2.E-08	European	^10^
	3q13.33	*TIMMDC1*/*TMEM39A*	rs2293370	0.16	1.39	7.E-16	European	^12^
			rs3732421	0.34	1.35	3.E-13	Chinese	^17^
			**rs57271503**	**0**.**34**	**0**.**72**	**3**.**E-09**	**Japanese**	**This study**
	3q25.33	*IL-12A*/*IL12A-AS1*/*IQCJ*/*SCHIP1*	rs2366643, etc.	0.38	0.62	3.E-35	European	^12^
			rs582537	0.29	1.33	2.E-11	Chinese	^17^
4	4p16.3	*DGKQ*	rs11724804	0.44	1.22	9.E-12	European	^14^
	4q24	*NFKB1*/*MANBA*	rs7665090	0.49	1.26	8.E-14	European	^12^
			rs1598856	0.49	1.26	2.E-10	Chinese	^17^
			**rs17033015**	**0**.**47**	**1**.**35**	**9**.**E-10**	**Japanese**	**This study**
	4q27	*IL21-AS1*	rs925550	0.35	1.31	4.E-13	Chinese	^17^
5	5p13.2	*IL7R*/*CAPSL*	rs6871748	0.28	1.30	2.E-13	European	^12^
			**rs12697352**	**0**.**19**	**0**.**68**	**2**.**E-09**	**Japanese**	**This study**
	5q21.1	*NUDT12*/*C5orf30*	rs526231	0.32	1.15	1.E-08	European	^14^
	5q33.3	*IL12B*	rs2546890	0.50	1.15	1E-10	European	^14^
6	6q23.3	*OLIG3*/*TNFAIP3*	rs6933404	0.17	1.18	1E-10	European	^14^
7	7p14.1	*ELMO1*	rs6974491	0.16	1.25	4.E-08	European	^10^
	7q32.1	*IRF5*/*TNPO3*	rs10488631	0.10	1.59	5.E-23	European	^14^
9	9q32	*TNFSF15*/*TNFSF8*	rs4979467	0.37	1.53	1.E-29	Chinese	^17^
			**rs4979462**	**0**.**32**	**0**.**60**	**2**.**E-26**	**Japanese**	**This study**
11	11q13.1	*RPS6KA4*	rs538147	0.39	1.23	2.E-10	European	^10^
	11q23.3	*DDX6*/*CXCR5*	rs80065107	0.19	1.39	7.E-16	European	^12^
			rs77871618	0.16	1.40	3.E-13	Chinese	^17^
12	12p13.31	*TNFRSF1A*	rs1800693	0.43	0.74	7.E-19	European	^12^
			rs4149576	0.14	1.37	4.E-09	Chinese	^17^
	12q24.12	*ATXN2*/*BRAP*	rs11065987	0.37	1.19	3.E-08	European	^14^
13	13q14.2	*DLEU1*/*BCMS*	rs9591325	0.05	1.63	1.E-10	European	^14^
14	14q24.1	*RAD51B*	rs911263	0.33	1.29	2.E-11	European	^10^
	14q32.32	*EXOC3L4*	rs2297067	0.22	1.39	6.E-19	European	^14^
15	15q25.1	*IL16*	rs11556218	0.19	1.29	9.E-09	Chinese	^17^
16	16p12.1	*IL21R*	rs2189521	0.30	1.41	4.E-16	Chinese	^17^
	16p13.13	*RMI2*/*CLEC16A*	rs1646019, etc.	0.30	1.38	2.E-23	European	^12^
	16q21	*CSNK2N2*/*CCDC113*	rs2550374	0.48	1.23	2.E-08	Chinese	^17^
	16q24.1	*IRF8*/*FOXF1*	rs11117432	0.23	1.31	5.E-11	European	^10^
17	17q21.1	*ZPBP2*/*GSDMB*/*IKZF3*	rs8067378	0.47	1.26	6.E-14	European	^12^
			rs9635726	0.41	1.37	2.E-16	Chinese	^17^
			**rs4795395**	**0**.**35**	**1**.**42**	**4**.**E-12**	**Japanese**	**This study**
	17q21.31	*MAPT*	rs17564829	0.23	1.25	2.E-09	European	^12^
19	19p13.2	*TYK2*	rs34536443	0.03	1.95	1.E-12	European	^12^
	19p13.3	*ARID3A*	rs10415976	0.47	1.30	4.E-12	Chinese	^17^
	19q13.33	*SPIB*	rs3745516	0.21	1.39	1.E-20	European	^14^
22	22q13.1	*SYNGR1*/*PDGFB*/*RPL3*	rs2267407	0.24	1.29	1.E-13	European	^12^
			rs137603	0.13	1.37	3.E-08	Chinese	^17^

^a^MAF (minor allele frequency) in 1000 genome project in each population.

^b^OR (odds ratio).

Thousands of genetic variations associated with susceptibility to human complex diseases have been identified by GWASs^18^. Among genes located near the “top-hit SNP” in each susceptibility locus, candidate genes with well-known functions are often selected as the “disease susceptibility genes”. The majority of SNPs that regulate gene expression are actually found in the vicinity of genes within 100 kb of the transcription start site (TSS)^19^. However, trans-acting expression quantitative trait loci (e-QTL) variations, whose target transcripts are separated by arbitrary distances, are believed to explain a substantial proportion of the heritable variation in gene expression^20^. For example, although *FTO* was reported as a susceptibility gene for obesity, the effector genes whose expression levels were influenced by the significantly associated SNPs were not *FTO* but *Iroquois Homeobox 3* (*IRX3*) and *IRX5*^21^. In PBC, a locus on chromosome 17q12-21 (*ORMDL3-GSDMB-ZPBP2-IKZF3*) has been reported as a shared susceptibility locus in different populations. Although the top-hit SNP was located in the *IKAROS family zinc finger 3* (*IKZF3*), in which the function of the protein product is related to the proliferation and differentiation of B cells, the effector gene in this locus was identified as *ORMDL sphingolipid biosynthesis regulator 3* (*ORMDL3*), whose protein product regulates endoplasmic reticulum (ER)-mediated Ca^2+^ homeostasis and facilitates the unfolded-protein response (UPR)^22,23^. Therefore, understanding the contribution of susceptibility loci to the onset of diseases requires identification of the effector genes that are regulated by the primary functional variation located in the disease susceptibility loci.

The present study aimed to further elucidate the genetic architecture of PBC in the Japanese population. To this end, we performed a GWAS and subsequent genome-wide meta-analysis based on a whole-genome SNP imputation analysis with previous GWAS^16^. The PBC susceptibility locus chromosome 3q13.33 (including *ARHGAP31*, *TMEM39A*, *POGLUT1*, *TIMMDC1*, and *CD80*) has been identified by GWAS as a PBC susceptibility locus in European and Chinese populations; consequently, this genome-wide meta-analysis involved replicating chromosome 3q13.33 in the Japanese population. Here, we show *in silico* and *in vitro* functional analyses and identify the effector gene and the primary functional SNP in the PBC susceptibility locus chromosome 3q13.33.

## Results

### GWAS and genome-wide meta-analysis

We genotyped an independent set of 1,148 samples (668 PBC cases and 480 healthy controls) using the Affymetrix Japonica V1 Array^24^. Thirty-four samples were excluded by Dish QC (<0.82) or overall call rate for a total of 20,000 SNPs (<0.97) and 13 samples were excluded because of cryptic relatives. A further 13 samples were located far from the JPT cluster drawn using the first and second components after PCA and were removed from further analysis (Supplementary Fig. 1A). We re-genotype called about 2,897 samples (1,392 PBC cases and 1,505 healthy controls) collected in the previous study^16^. Eighteen samples were excluded by Dish QC (<0.82) or overall call rate for a total of 20,000 SNPs (<0.97) and 15 samples were excluded because of cryptic relatives. Seventeen samples were located far from the HapMap JPT cluster drawn using the first and second components after PCA and were removed from further analysis (Supplementary Fig. 1B).

A quantile-quantile plot of the distribution of test statistics for the comparison of allele frequencies in the PBC cases and healthy controls provided an inflation factor lambda value of 1.097 for all tested SNPs for the 1,148 entries in the current dataset and a value 1.061 for the 2,897 entries in the previous dataset (Supplementary Fig. 2). Genotype imputation and the association study were separately performed for the two datasets. The process of data cleaning and meta-analysis is summarized in Supplementary Fig. 3.

**Figure 1:**
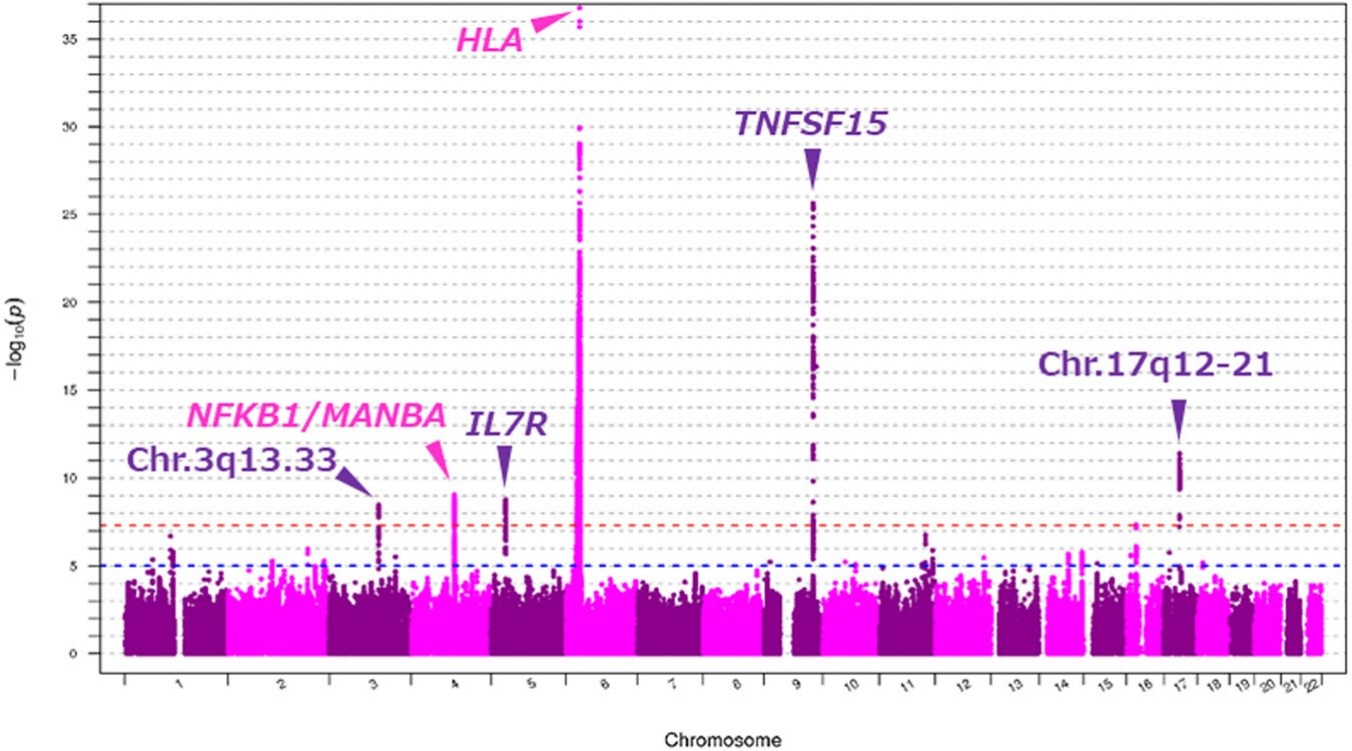
Genome-wide Manhattan plot of the GWAS meta-analysis between two data sets from the ASI and Japonica platforms. Negative log 10 P-values of qualified SNPs are plotted against their genomic positions. The genome-wide significance line (red) is shown at 7.30 (−log10 P [5e-8]). The genome-wide suggestive line (blue) is shown at 5 (−log10 P [1e-5]).

**Figure 2:**
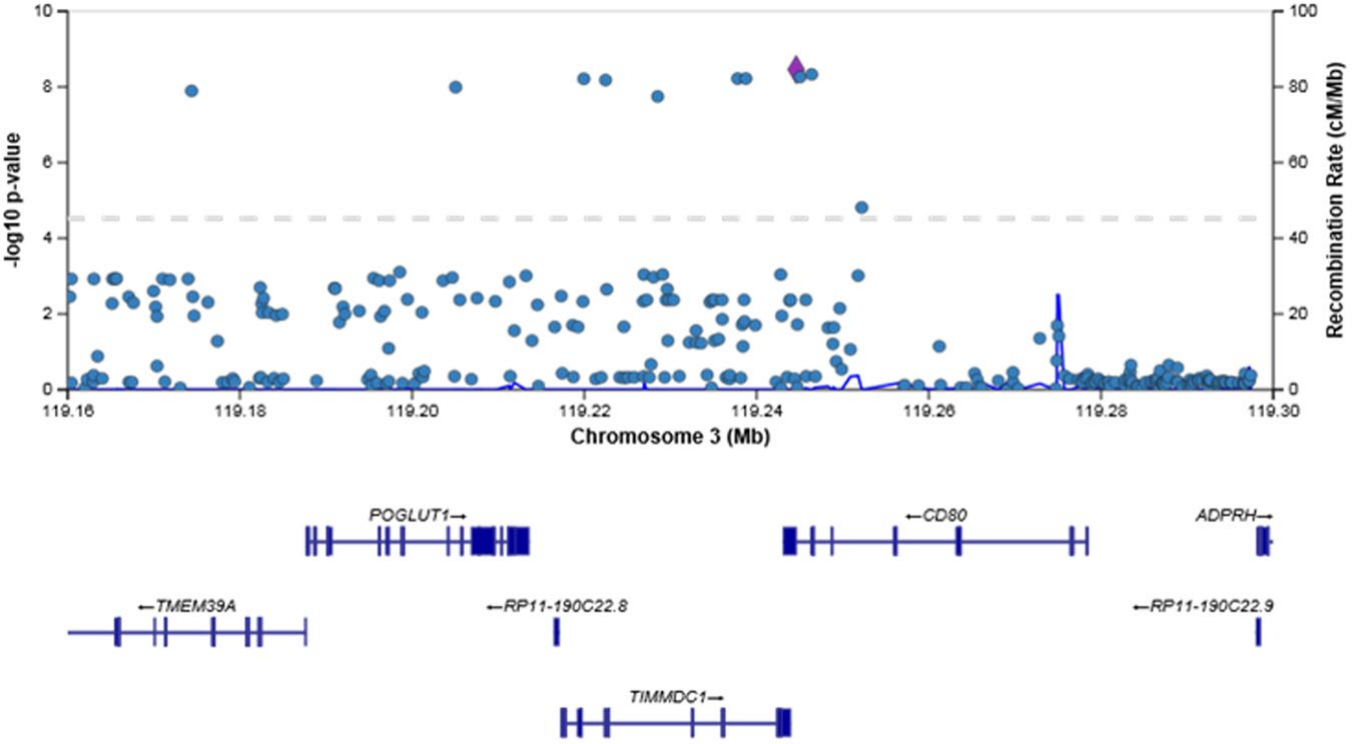
Regional plot of association results and recombination rates for the region surrounding *POGLUT1* (chromosome 3: 119,160,000-119,300,300). Each dot shows the P-value of each SNP after meta-analysis. The purple diamond represents the SNP with the minimum P-value in the region. Genetic recombination rates are shown with a blue line.

Figure 1 shows a genome-wide view of the single-point association data based on allele frequencies after meta-analysis. The loci *HLA*, *TNFSF15*, *IL7R*, *NFKB1*/*MANBA*, and chromosome 17q12-21 showed significant associations with PBC, as reported in the previous GWAS performed on a Japanese population^16^. In addition to these regions, meta-analysis to combine the two datasets showed a significant association in chromosome 3q13.33 (Top hit SNP: rs57271503, OR = 0.7241, *P* = 3.5 × 10^−9^, Fig. 2), although this locus appears as evidence of no association with PBC from studies using each platform (Japonica and ASI, Supplementary Fig. 4).

### Identification of rs2293370 as the primary functional SNP in chromosome 3q13.33

Among the 29 SNPs whose P values were less than 1.0 × 10^−6^ upon genome-wide meta-analysis, SNPs located in the 3′-untranslated region (UTR) and synonymous substitutions were selected as potential candidates for primary functional variation in the chromosome 3q13.33 region (Table 2 and Fig. 2). Five of the 29 SNPs [rs57271503 and rs3830649 in the 3′UTR of *CD80*, rs2305249 in exon 11 of *ARHGAP31* (P567P), rs1131265 in exon 3 of *TIMMDC1* (V146V), and rs3732421 in the 3′UTR of *TMEM39A*] are located in the 3′UTR or synonymous substitutions but are unrelated to their own gene expression as determined by e-QTL analysis^25^ (Supplementary Fig. 5) and thus were excluded from further analysis.

**Table 2:** 29 SNPs associated with susceptibility to PBC in the Japanese population in chromosome 3q13.33 by high-density association mapping.

rs number	SNP location (Chr.3)	GWAS/imputation	Allele 1	Allele 2	P value	OR	Regulome DB score	UCSC (Regulatory Motifs)	Location
rs57271503	119244593	imputation	G	A	3.48E-09	0.724	No data	×	*CD80* 3′UTR
rs9855065	119130141	imputation	G	A	3.57E-09	0.725	No data	Δ	*ARHGAP31* intron 11
rs3830649	119246385	imputation	G	del	4.66E-09	0.727	No data	×	*CD80* 3′UTR
rs2305249	119128398	imputation	G	A	5.07E-09	0.727	6	×	*ARHGAP31* Exon 11 (P567P)
rs13092998	119245044	GWAS (Japonica)	G	T	5.45E-09	0.728	No data	×	*CD80* intron 6
rs62264485	119237798	imputation	C	A	6.00E-09	0.728	6	Δ	*TIMMDC1* intron 6
rs35264490	119238753	imputation	A	del	6.00E-09	0.728	No data	Δ	*TIMMDC1* intron 6
**rs2293370**	**119219934**	**GWAS (ASI, Japonica)**	**G**	**A**	**6.08E-09**	**0.728**	**3a**	**Δ**	***TIMMDC1*** **intron 2**
rs1463138	119128634	imputation	T	C	6.34E-09	0.751	6	×	*ARHGAP31* intron 11
rs1131265	119222456	imputation	G	C	6.57E-09	0.729	No data	×	*TIMMDC1* Exon 3 (V146V)
rs1463139	119128628	imputation	A	G	6.70E-09	0.749	No data	×	*ARHGAP31* intron 11
rs3732421	119150089	imputation	A	G	9.93E-09	0.732	5	×	*TMEM39A* 3′UTR
rs7650774	119205050	imputation	T	C	1.01E-08	0.731	No data	×	*POGLUT1* intron 6
rs12636784	119174383	imputation	A	G	1.27E-08	0.733	6	×	*TMEM39A* intron 3
rs4687853	119130360	imputation	A	G	1.47E-08	0.754	6	×	*ARHGAP31* intron 11
rs9843355	119228508	imputation	G	A	1.79E-08	0.735	6	×	*TIMMDC1* intron 4
rs1530687	119114515	imputation	G	A	6.26E-08	0.767	5	Δ	*ARHGAP31* intron 8
rs9831023	119111762	GWAS (ASI, Japonica)	T	C	6.98E-08	0.768	5	×	*ARHGAP31* intron 7
rs9884090	119116150	imputation	G	A	9.34E-08	0.753	5	×	*ARHGAP31* intron 8
rs1000198	119113820	imputation	A	C	1.34E-07	0.771	6	×	*ARHGAP31* intron 8
rs11922594	119125822	imputation	T	C	1.50E-07	0.771	5	×	*ARHGAP31* intron 10
rs6773050	119123814	GWAS(ASI)	C	T	1.62E-07	0.774	6	×	*ARHGAP31* intron 10
rs12494314	119122820	imputation	T	C	1.90E-07	0.758	No data	×	*ARHGAP31* intron 10
rs4279094	119114693	GWAS(ASI)	A	G	2.12E-07	0.774	4	×	*ARHGAP31* intron 8
rs9846036	119116064	imputation	A	C	2.12E-07	0.774	5	×	*ARHGAP31* intron 8
**rs56008261**	**119114927**	**GWAS(Japonica)**	**T**	**C**	**2.61E-07**	**0.776**	**1b**	**O**	***ARHGAP31*** **intron 8**
rs6776377	119115556	imputation	T	C	2.81E-07	0.776	5	×	*ARHGAP31* intron 8
rs6787836	119115567	imputation	A	G	5.85E-07	0.782	5	×	*ARHGAP31* intron 8
rs11715698	119118497	imputation	A	G	6.61E-07	0.788	5	×	*ARHGAP31* intron 9

Two of the remaining 24 SNPs had RegulomeDB scores higher than 3 and these scores were supported by their location in DNase hyper-sensitivity clusters and the binding of transcription factor. Consequently, these two SNPs were selected as potential candidates^26^ (Table 2; rs2293370 in intron 2 of *TIMMDC1* and rs56008261 in intron 8 of *ARHGAP31*). Both SNPs were located in DNase I hyper-sensitivity clusters and in H3K27Ac markers in at least one cell type identified by the UCSC genome browser^27^.

**Figure 3:**
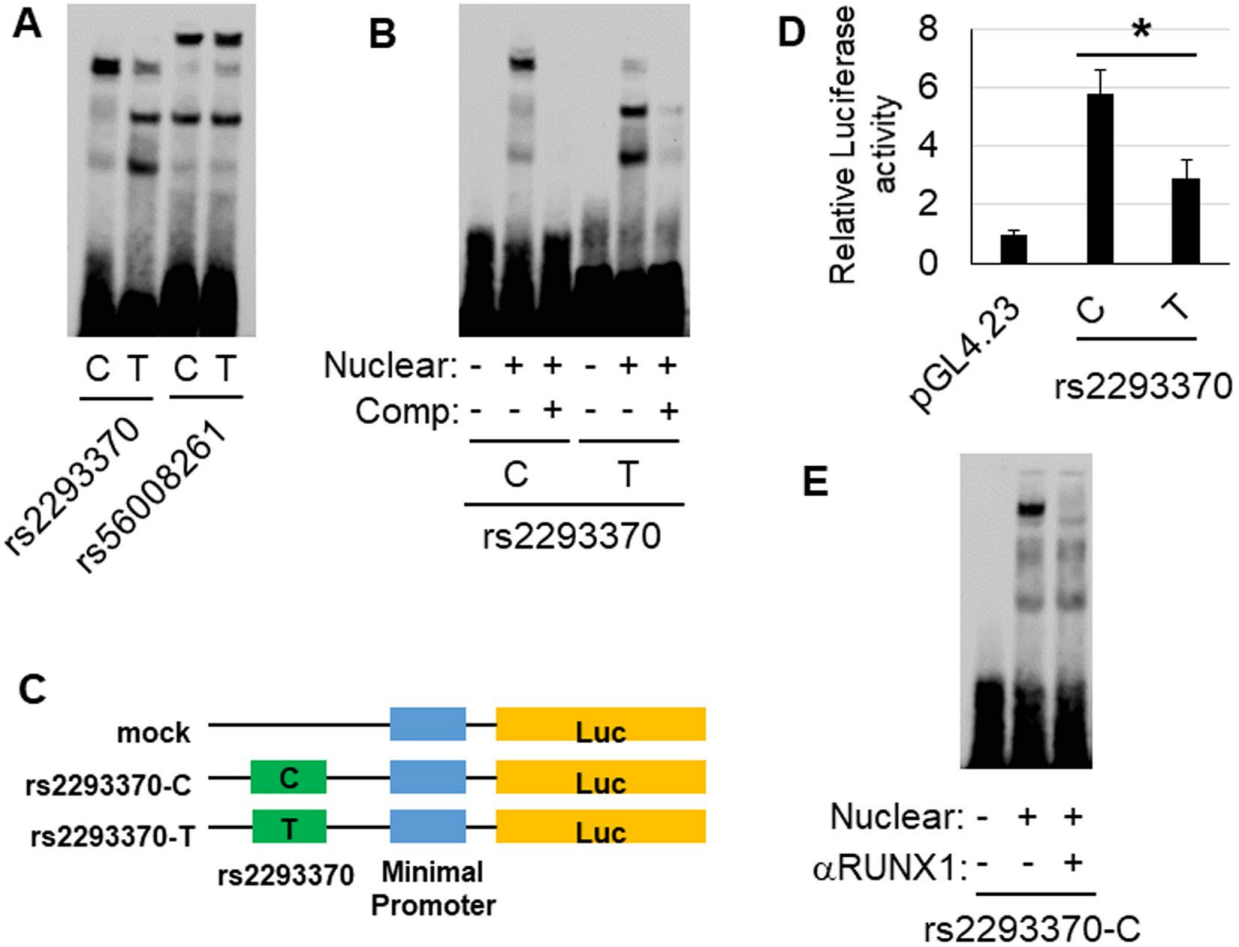
*In vitro* functional analysis of each candidate variation in chromosome 3q13.33. (**A**) EMSA of each candidate primary variation using biotin-labeled probes corresponding to the major and the minor allele, and nuclear extracts of HepG2 cells. rs2293370 was the only SNP to show a difference in mobility shift between the two alleles. (**B**) Competitor assay, using HepG2 nuclear extracts and a 200× concentration of unlabeled probe corresponding to either the C (i.e., PBC susceptibility) or T alleles of rs2293370. (**C**) Outline of reporter plasmid constructs. PCR fragments of intron 2 of *TIMMDC1*, containing rs2293370, were sub-cloned into the pGL4.23 vector. (**D**) Transcription was measured by cellular luciferase activity, 24 h after transfection of HepG2 cells. The luciferase activities of cells transfected with the PBC susceptibility allele (C allele) of rs2293370 were higher than those transfected with the T allele. Three independent experiments with triplicate measurements were performed for each assay, and data represent mean ± SD; *P < 0.05 (Student’s *t*-test). (**E**) Identification of transcription factors targeting the C allele of rs2293370. A super-shift was observed following incubation of HepG2 cell nuclear extracts with an anti-RUNX1 antibody. Three independent experiments were performed in each assay.

We performed electrophoretic mobility shift assays (EMSAs) to evaluate the effect of candidate SNPs that potentially regulate the binding affinity of transcription factors. A difference in mobility shift between the major allele and the minor allele was detected for rs2293370 in HepG2 (Fig. 3A) and Jurkat (Supplementary Fig. 6A) cells. The shifted band was abrogated by incubation with a 200× concentration of a non-labeled probe (competitor probe) (Fig. 3B and Supplementary Fig. 6B). In contrast, there was no difference in mobility shift for rs56008261 between the major allele and the minor allele (Fig. 3A and Supplementary Fig. 6A).

Additionally, in order to deny the possibility of the existence of other variations with independent genetic contributions in chromosome 3q13.33, conditional logistic regression analysis was performed. When the rs2293370 was conditioned on, significant associations of other SNPs in chromosome 3q13.33 totally disappeared (Supplementary Fig. 7).

These results indicated that rs2293370 is the primary functional SNP in chromosome 3q13.33.

### Molecular features of disease susceptibility influenced by rs2293370

We performed luciferase reporter assays in HepG2 and Jurkat cells to determine the differences in transcription efficiency between the C (major allele, PBC-susceptibility) and T (minor allele, PBC-protective) alleles of rs2293370. Concordant with the result of EMSA, the luciferase activity of cells 24 h after transfection with a reporter construct containing the C allele of rs2293370 was significantly higher than that of cells containing the T allele for both cell lines (Fig. 3C,D and Supplementary Fig. 6C).

Next, we performed *in silico* prediction of transcription factor binding using the TRANSFAC database^28^ to identify the transcription factor responsible for the mobility shift associated with the C allele of rs2293370. The C allele of rs2293370, but not the T allele, was predicted to be involved in a binding motif of Runt-related transcription factors (Supplementary Fig. 8). Although the DMRT and Myb families also showed similar patterns, they are not expressed in HepG2 or Jurkat cells^29^. Of the Runt-related transcription factors, Runt-related transcription factor (RUNX1) -1, but not RUNX-2 and RUNX-3, was confirmed to be expressed in both HepG2 and Jurkat cells^29^ (Supplementary Fig. 9). Consistent with the *in silico* prediction of transcription factor binding, the mobility shift associated with the C allele of rs2293370 was reduced by pre-incubation with an anti-RUNX1 antibody prior to electrophoresis (Fig. 3E).

These results indicated that the PBC susceptibility allele of rs2293370 enhances transcription via binding with RUNX1.

### The mRNA expression level of POGLUT1 is influenced by rs2293370

**Figure 4:**
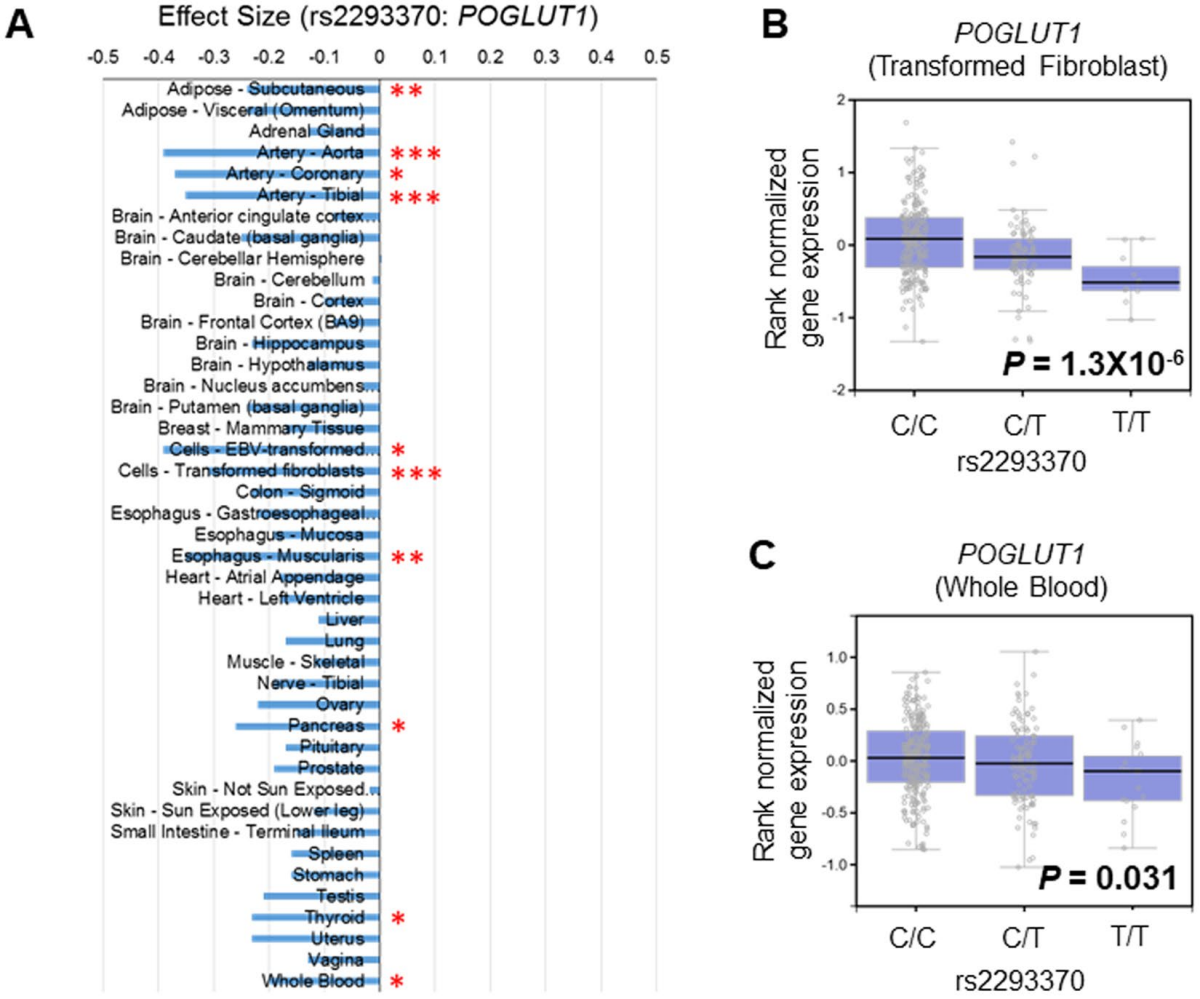
rs2293370 genotypes are associated with differences in endogenous *POGLUT1* expression levels. (**A**) The relationship between rs2293370 genotype and the endogenous expression of *POGLUT1* was compared in all tissues registered in the GTEX database. The effect sizes of the rs2293370 minor allele (T allele; disease protective) in every organ are shown. Statistical significance levels before Bonferroni multiple comparison correction were *P* = 0.00116. *Pc < 0.05, **Pc < 0.005, and ***Pc < 0.0005, after Bonferroni multiple comparison correction. (**B** and **C**) Box plots showing the association between endogenous *POGLUT1* expression and rs2293370 genotypes in (**B**). Transformed Fibroblast and (**C**) Whole Blood.

We used the GTEx portal database^25^ to assess the influence of rs2293370 on endogenous gene expression by comparing the expression levels of all genes in the human genome for the different genotypes of rs2293370 in every organ whose expression level of *POGLUT1* was above the threshold for detection. Individuals carrying the C allele (i.e., the PBC-susceptible allele) of rs2293370 showed a significantly higher level of expression of *POGLUT1* in several organs (Fig. 4; statistical significance level: *P* < 0.05/47 organs = 0.00106). Other genes located in chromosome 3q13.33 (*ARHGAP31*, *TMEM39A*, *POGLUT1*, *TIMMDC1*, and *CD80*) showed no significant association between rs2293370 genotypes and gene expression (Supplementary Fig. 10).

## Discussion

In the present study we identified chromosome 3q13.33, which includes the genes *ARHGAP31*, *TMEM39A*, *POGLUT1*, *TIMMDC1*, and *CD80*, as a PBC susceptibility locus in the Japanese population by genome-wide meta-analysis based on whole-genome SNP imputation analysis of two distinct data sets of Japanese PBC-GWAS. The role of chromosome 3q13.33 had previously been identified in European and Chinese populations. Consequently, rs2293370, which is located in intron 2 of *TIMMDC1*, was identified as the primary functional SNP for disease susceptibility to PBC in chromosome 3q13.33 by *in silico* and *in vitro* functional analyses. In addition, the disease protective allele of rs2293370 was shown to disrupt a RUNX1 binding site and was associated with significantly decreased *POGLUT1* mRNA expression levels in tissues compared with individuals without this allele.

The contribution of *POGLUT1* to the pathogenesis of PBC has not been reported to date. Endoplasmic reticulum (ER)-localized protein O-glucosyltransferase 1, which is encoded by *POGLUT1*, adds glucose moieties to serine residues of the epidermal growth factor (EGF)-like domains of Notch family proteins^30,31^. Notch signaling is an evolutionally conserved cascade that includes four receptors (Notch 1–4) and five ligands [Jagged 1, Jagged 2, Delta-like ligand 1 (DLL1), DLL3 and DLL4]. Therefore, it might be possible that genetic polymorphisms affecting the expression levels of *POGLUT1* influence the Notch signaling pathway by altering Notch glycosylation. The generation and development of diverse blood cell lineages and peripheral immune responses are regulated by this Notch signaling cascade, especially in hematopoiesis during T cell lineage commitment and maturation in the thymus, and during marginal zone B (MZB) cell development in the spleen^32^. Recently, dendritic cell (DC) homeostasis and the development of several lymphocyte subsets belonging to the innate immune system have been reported to be regulated by Notch^32^. Therefore, inappropriate immune responses against self-antigens could occur due to impaired regulation of Notch signaling. In experimental autoimmune encephalomyelitis (EAE) and non-obese diabetic (NOD) mice, which are mouse models for multiple sclerosis and type 1 diabetes (T1D), respectively, disease progression was impeded by the administration of blocking antibodies against Notch receptors or DLL4^32^. Therefore, higher endogenous levels of *POGLUT1* caused by the PBC-susceptible allele of rs2293370 may induce excessive Notch signaling and inappropriate immune responses against self-antigens. Very importantly, Notch signaling is also involved in the development or formation of intrahepatic bile ducts. Mutations in *JAG1* or *Notch2* are known causes of Alagille syndrome, an autosomal dominant disease characterized by congenital cholangiopathy with jaundice and bile duct paucity^33–35^. *POGLUT1* was shown to regulate the number of bile ducts around portal veins in a JAG1-dependent manner using *JAG1*^+/−^ and *POGLUT1*^+/−^ mice^36^. These results indicate that *POGLUT1* might be involved in the mechanism of bile duct loss in PBC. However, a limitation of this study was that endogenous *POGLUT1* expression levels in PBC patients were not examined in this study. Additional studies are warranted to improve our understanding of the relationship between PBC pathogenesis and *POGLUT1*.

A more than 100-kb stretch of the genome is located in chromosome 3q13.33 that includes *ARHGAP31*, *TMEM39A*, *POGLUT1*, *TIMMDC1*, and *CD80*. In addition to the present study on the Japanese population, this locus, as represented by top-hit SNP rs2293370, has been reported as a susceptibility region to PBC in European and Chinese populations^10,14,17^. The present study identified rs2293370 as the primary functional SNP by *in silico* and *in vitro* functional analysis. Therefore, *POGLUT1* is likely the effector gene for susceptibility to PBC in not only the Japanese population but also other populations. This locus has also been reported as a susceptibility region for celiac disease, multiple sclerosis, systemic lupus erythematosus, and vitiligo, represented by rs11712165 in *ARHGAP31*, rs2293370 in *TIMMDC1*, and rs6804441 and rs148136154 in *CD80*, respectively^37–40^. The LDs were not strong between these represented tag-SNPs and rs2293370, which was identified as the primary functional SNP in this study (r^2^ < 0.4 in 1000 genomes Asian, Supplementary Fig. 11). Regardless, rs2293370, whose effector gene is *POGLUT1*, may operate as a functional SNP in both PBC and multiple sclerosis.

The present study, compared with the GWAS in the European descent, identified four out of 30 non-*HLA* loci (*IL7R*, *NFKB1*/*MANBA*, chromosome 17q12-21, and chromosome 3q13.33) as susceptibility loci in the Japanese population. Additionally, even though the p-values were below the genome-wide significance level (*P* < 5.0 × 10^−8^), the direction of OR was the same between a European descent population and the Japanese population in *MMEL1*, *STAT4*/*STAT1*, *IL12A*, *CXCR5*/*DDX6*, and *SPIB* (Table 1). These loci are likely candidates as shared susceptibility loci for the pathogenesis of PBC between European and Japanese populations. We are currently working to clarify the shared gene profiles between different populations by conducting SNP imputation and subsequent meta-analysis using GWAS data from an international collaboration involving the UK, Italy, the USA, Canada, China and Japan.

In conclusion, genome-wide meta-analysis together with *in silico*/*in vitro* functional analyses identified the primary functional SNP rs2293370 and the effector gene *POGLUT1*. Chromosome 3q13.33 contains *CD80*, which encodes a well-known co-stimulatory signaling molecule necessary for antigen presentation from HLA class II to T cell receptor (TCR). *CD80* had been assumed as a candidate effector gene in previous GWASs, whereas our approach identified *POGLUT1* as the target transcript for disease susceptibility in this locus comprehensively without stereotypes. Of the PBC susceptibility genes identified in the Japanese population, we previously identified the primary functional SNPs of *TNFSF15*, *PRKCB*, and chromosome 17q12-21, and their effector genes^16,22,41^, as well as chromosome 3q13.33 in the present study. Similar post-GWAS approaches for susceptibility genes are needed to further clarify the molecular mechanisms of disease development.

## Materials and Methods

### Subjects

DNA samples for GWAS using the Japonica array platform were collected from 1,148 individuals (668 PBC cases and 480 healthy controls). The demographics of the PBC cases and controls are shown in Supplementary Table 1.

Written informed consent was obtained from all participants. The protocol of this study was approved by the committee on research ethics and genetically modified organisms of the Graduate School of Medicine, The University of Tokyo, by the ethics committees of Nagasaki Medical Center, and by the ethics committees of Tohoku Medical Megabank Organization, Tohoku University. All experiments were performed in accordance with relevant guidelines and regulations.

### Genotyping, quality control, and genotype imputation

Genotyping was performed using the Japonica V1 array (1,148 Japanese individuals in the present study; Affymetrix Japan). Genotype calling was performed with the apt-probeset-genotype program in Affymetrix Power Tools ver. 1.18.2 (Thermo Fisher Scientific Inc., Waltham, MA). Sample quality control was conducted by following the manufacturer’s recommendation (dish QC > 0.82 and sample call rate > 97%). Clustering of each SNP was evaluated by the Ps classification function in the SNPolisher package (version 1.5.2, Thermo Fisher Scientific Inc.). SNPs that were assigned “recommended” by the Ps classification function were used for downstream analyses. SNPs that satisfied the following criteria were used for genotype imputation: a call rate > 99.0%, a Hardy-Weinberg Equilibrium (HWE) p-value > 0.0001, and a minor allele frequency (MAF) > 0.5%. Pre-phasing was conducted with EAGLE v2.3.2^42^ with default settings. Genotype imputation was conducted with IMPUTE4 v1.0^43^ using a phased reference panel of 2,049 Japanese individuals from a prospective, general population cohort study performed by the Tohoku Medical Megabank Organization (ToMMo)^44,45^. These procedures were conducted using default settings. Cryptic relatives were excluded using PRIMUS^46^ with default settings. In addition, principal component analysis (PCA) was performed using East Asian samples from the International 1000 Genome Project (104 JPT, 103 CHB, 93 CHS, 91 CDX, and 99 KHV samples) in addition to the case and control samples. PCA identified outliers to be excluded using the Smirnov-Grubbs test with a Bonferroni corrected p-value < 0.05. We had previously analyzed the data (2,897 Japanese individuals) using the Axiom Genome-Wide ASI 1 Array^16^ and re-analyzed the data using the above-mentioned procedures.

### Association analysis and meta-analysis

Association analysis was performed with PLINK (version 1.9) in each dataset (i.e., 2,897 ASI array data and 1,148 Japonica array data). The following options were used for PLINK: a call rate > 97.0%, a HWE p-value > 0.000001, a minor allele frequency (MAF) > 0.1%, and a logistic regression model.

Meta-analysis was performed using PLINK with the meta-analysis option after excluding duplicates between the two datasets. The fixed-effects meta-analysis p-value was used.

### Databases

The probability that a candidate functional variation might influence transcription regulation was evaluated using the RegulomeDB database (http://www.regulomedb.org/index)^26^ and the UCSC genome browser (http://genome.ucsc.edu/index.html)^27^. Transcription factor binding was predicted using TRANSFAC Professional (QIAGEN, Valencia CA; http://www.gene-regulation.com/pub/databases.html)^28^. Gene expression levels in each cell line and the correlation between the genotypes of candidate SNPs and gene expression were examined using GeneCards (http://www.genecards.org/) and the GTEx portal database (http://gtexportal.org/home/), respectively^25,29^. P values < 0.05, after adjustment for multiple testing (Bonferroni correction), were regarded as statistically significant.

### Electrophoretic mobility shift assay (EMSA)

EMSA was performed using a LightShift Chemiluminescent EMSA Kit (Thermo-Fisher Scientific) and biotin-labeled double-stranded oligonucleotide probes corresponding to each major and minor allele (Supplementary Table 2), according to the manufacturer’s instructions. These oligonucleotide probes (10 fmol/μL) and a nuclear extract (2.5 μg/mL) of HepG2 or Jurkat cells (Nuclear Extract Kit; Active Motif, Carlsbad, CA) were incubated together for 30 min at 25 °C.

The super-shift assay was performed by incubating the biotin-labeled probe with the nuclear extracts for 30 min at 25 °C, before subsequently incubating these complexes with Anti-RUNX1/AML1 antibody - ChIP Grade (ab23980) (Abcam, Cambridge, UK) for 30 min at 25 °C.

Each assay was independently performed three times.

### Luciferase reporter assay

Amplicons of part of the 2nd intron of *TIMMDC1*, which contain each allele of rs2293370, were obtained from human genomic DNA using specific PCR primers (Supplementary Table 3) and were then subcloned into the luciferase reporter pGL4.23 (luc2/minP) vector (Promega, Madison, WI). pGL4.23 constructs (500 ng) of each allele and 50 ng of the pGL4.74 (hRluc/TK) vector as an internal control were transfected into Jurkat and HepG2 cells using Lipofectamine 3000 (Thermo-Fisher Scientific). The Dual-Luciferase Reporter Assay system (Promega) was used to measure luciferase activity. Differences in relative luciferase activity were compared between the major and minor alleles of each SNP using Student’s *t*-test. P values < 0.05 were regarded as statistically significant. Each figure shows representative data from experiments performed independently three times. The data in the figures represent the mean ± standard deviation of triplicate assays in a single experiment.

## Electronic supplementary material

Supplementary data

## References

[1] Kaplan MM, Gershwin ME (2005). Primary biliary cholangitis. N. Engl. J. Med..

[2] Selmi C, Bowlus CL, Gershwin ME, Coppel RL (2011). Primary biliary cirrhosis. Lancet.

[3] Gershwin ME, Mackay IR (2008). The causes of primary biliary cirrhosis: convenient and inconvenient truths. Hepatology.

[4] Shimoda S (2015). Natural killer cells regulate T cell immune responses in primary biliary cirrhosis. Hepatology.

[5] Jones DE, Watt FE, Metcalf JV, Bassendine MF, James OF (1999). Familial primary biliary cholangitis reassessed: a geographicallybased population study. J. Hepatol..

[6] Selmi C (2004). Primary biliary cholangitis in monozygotic and dizygotic twins: genetics, epigenetics, and environment. Gastroenterology.

[7] Hirschfield GM (2009). Primary biliary cholangitis associated with HLA, IL12A, and IL12RB2 variants. N. Engl. J. Med..

[8] Hirschfield GM (2010). Variants at IRF5-TNPO3, 17q12-21 and MMEL1 are associated with primary biliary cholangitis. Nat. Genet..

[9] Liu X (2010). Genome-wide meta-analyses identify three loci associated with primary biliary cholangitis. Nat. Genet..

[10] Mells GF (2011). Genome-wide association study identifies 12 new susceptibility loci for primary biliary cholangitis. Nat. Genet..

[11] Hirschfield GM (2012). Association of primary biliary cholangitis with variants in the CLEC16A, SOCS1, SPIB and SIAE immunomodulatory genes. Genes Immun..

[12] Liu JZ (2012). Dense fine-mapping study identifies new susceptibility loci for primary biliary cholangitis. Nat. Genet..

[13] Juran BD (2012). Immunochip analyses identify a novel risk locus for primary biliary cirryosis at 13q14, multiple independent associations at four established risk loci and epistasis between 1p31 and 7q32 risk variants. Hum. Mol. Genet..

[14] Cordell HJ (2015). International genome-wide meta-analysis identifies new primary biliary cholangitis risk loci and targetable pathogenic pathways. Nat. Commun..

[15] Nakamura M (2012). Genome-wide association study identified TNFSF15 and POU2AF1 as susceptibility locus for primary biliary cholangitis in the Japanese population. Am. J. Hum. Genet..

[16] Kawashima M (2017). Genome-wide association study identified PRKCB as a genetic susceptibility locus for primary biliary cholangitis in a Japanese population. Hum. Mol. Genet..

[17] Qiu F (2017). A genome-wide association study identifies six novel risk loci for primary biliary cholangitis. Nat. Commun..

[18] Welter D (2014). The NHGRI GWAS Catalog, a curated resource of SNP-trait associations. Nucleic Acids Res..

[19] Montgomery SB, Dermitzakis ET (2011). From expression QTLs to personalized transcriptomics. Nat. Rev. Genet..

[20] Cheung VG (2010). Polymorphic cis- and trans-regulation of human gene expression. Plos Biol..

[21] Claussnitzer M (2015). FTO Obesity Variant Circuitry and Adipocyte Browning in Humans. N. Engl. J. Med..

[22] Hitomi Y (2017). Identification of the functional variant driving ORMDL3 and GSDMB expression in human chromosome 17q12-21 in primary biliary cholangitis. Sci. Rep..

[23] Cantero-Recasens G, Fandos C, Rubio-Moscardo F, Valverde MA, Vicente R (2010). The asthma-associated ORMDL3 gene product regulates endoplasmic reticulum-mediated calcium signaling and cellular stress. Hum. Mol. Genet..

[24] Kawai Y (2015). Japonica array: improved genotype imputation by designing a population-specific SNP array with 1070 Japanese individuals. J. Hum. Genet..

[25] GTEx Consortium (2013). The Genotype-Tissue Expression (GTEx) project. Nat. Genet..

[26] Boyle AP (2012). Annotation of functional variation in personal genomes using RegulomeDB. Genome Res..

[27] Kent WJ (2002). The human genome browser at UCSC. Genome Res..

[28] Wingender E, Dietze P, Karas H, Knüppel R (1996). TRANSFAC: a database on transcription factors and their DNA binding sites. Nucleic Acids Res..

[29] Rebhan M, Chalifa-Caspi V, Prilusky J, Lancet D (1997). GeneCards: integrating information about genes, proteins and diseases. Trends Genet..

[30] Acar M (2008). Rumi is a CAP10 domain glycosyltransferase that modifies Notch and is required for Notch signaling. Cell.

[31] Moloney DJ (2000). Mammalian Notch1 is modified with two unusual forms of O-linked glycosylation found on epidermal growth factor-like modules. J. Biol. Chem..

[32] Radtke F, MacDonald HR, Tacchini-Cottier F (2013). Regulation of innate and adaptive immunity by Notch. Nat. Rev. Immunol..

[33] Li L (1997). Alagille syndrome is caused by mutations in human Jagged1, which encodes a ligand for Notch1. Nat. Genet..

[34] Oda T (1997). Mutations in the human Jagged1 gene are responsible for Alagille syndrome. Nat. Genet..

[35] McDaniell R (2006). NOTCH2 mutations cause Alagille syndrome, a heterogeneous disorder of the notch signaling pathway. Am. J. Hum. Genet..

[36] Thakurdas SM (2016). Jagged1 heterozygosity in mice results in a congenital cholangiopathy which is reversed by concomitant deletion of one copy of Poglut1 (Rumi). Hepatology.

[37] Dubois PC (2010). Multiple common variants for celiac disease influencing immune gene expression. Nat. Genet..

[38] International Multiple Sclerosis Genetics Consortium (2011). Genetic risk and a primary role for cell-mediated immune mechanisms in multiple sclerosis. Nature.

[39] Yang W (2013). Meta-analysis followed by replication identifies loci in or near CDKN1B, TET3, CD80, DRAM1, and ARID5B as associated with systemic lupus erythematosus in Asians. Am. J. Hum. Genet..

[40] Jin Y (2016). Genome-wide association studies of autoimmune vitiligo identify 23 new risk loci and highlight key pathways and regulatory variants. Nat. Genet..

[41] Hitomi Y (2015). Human primary biliary cirrhosis-susceptible allele of rs4979462 enhances TNFSF15 expression by binding NF-1. Hum. Genet..

[42] Loh PR (2016). Reference-based phasing using the Haplotype Reference Consortium panel. Nat. Genet..

[43] Bycroft, C. *et al*. Genome-wide genetic data on ~500,000 UK Biobank participants. *bioRxiv*, 166298 (2017).

[44] Nagasaki M (2015). Rare variant discovery by deep whole-genome sequencing of 1,070 Japanese individuals. Nat. Commun..

[45] Yamaguchi-Kabata Y (2015). iJGVD: an integrative Japanese genome variation database based on whole-genome sequencing. Hum. Genome Var..

[46] Staples J (2014). PRIMUS: rapid reconstruction of pedigrees from genome-wide estimates of identity by descent. Am. J. Hum. Genet..

