# Supplementary material for: POGLUT1, the putative effector gene driven by rs2293370 in primary biliary cholangitis susceptibility locus chromosome 3q13.33
Source: Sci Rep. 2019 Jan 14;9:102. doi: 10.1038/s41598-018-36490-1 (PMC6331557; doi:10.1038/s41598-018-36490-1)
Supplement: Supplementary file 1 — Supplementary data [file 41598_2018_36490_MOESM1_ESM.pdf]

Supplementary data to:

POGLUT1, the putative effector gene driven by rs2293370 in primary biliary cholangitis susceptibility locus chromosome 3q13.33

Yuki Hitomi[1,†], Kazuko Ueno[2,3,†], Yosuke Kawai[1], Nao Nishida[4], Kaname Kojima[2,3], Minae Kawashima[5], Yoshihiro Aiba[6], Hitomi Nakamura[6], Hiroshi Kouno[7], Hirotaka Kouno[7], Hajime Ohta[7], Kazuhiro Sugi[7], Toshiki Nikami[7], Tsutomu Yamashita[7], Shinji Katsushima[7], Toshiki Komeda[7], Keisuke Ario[7], Atsushi Naganuma[7], Masaaki Shimada[7], Noboru Hirashima[7], Kaname Yoshizawa[7], Fujio Makita[7], Kiyoshi Furuta[7], Masahiro Kikuchi[7], Noriaki Naeshiro[7], Hironao Takahashi[7], Yutaka Mano[7], Haruhiro Yamashita[7], Kouki Matsushita[7], Seiji Tsunematsu[7], Iwao Yabuuchi[7], Hideo Nishimura[7], Yusuke Shimada[7], Kazuhiko Yamauchi[7], Tatsuji Komatsu[7], Rie Sugimoto[7], Hironori Sakai[7], Eiji Mita[7], Masaharu Koda[7], Yoko Nakamura[7], Hiroshi Kamitsukasa[7], Takeaki Sato[7], Makoto Nakamuta[7], Naohiko Masaki[7], Hajime Takikawa[8], Atsushi Tanaka[8], Hiromasa Ohira[9], Mikio Zeniya[10], Masanori Abe[11], Shuichi Kaneko[12], Masao Honda[12], Kuniaki Arai[12], Teruko Arinaga-Hino[13], Etsuko Hashimoto[14], Makiko Taniai[14], Takeji Umemura[15], Satoru

Joshita[15], Kazuhiko Nakao[16], Tatsuki Ichikawa[16], Hidetaka Shibata[16], Akinobu Takaki[17], Satoshi Yamagiwa[18,33], Masataka Seike[19], Shotaro Sakisaka[20], Yasuaki Takeyama[20], Masaru Harada[21], Michio Senju[21], Osamu Yokosuka[22], Tatsuo Kanda[22], Yoshiyuki Ueno[23], Hirotoshi Ebinuma[24], Takashi Himoto[25], Kazumoto Murata[4], Shinji Shimoda[26], Shinya Nagaoka[6], Seigo Abiru[6], Atsumasa Komori[6,27], Kiyoshi Migita[6,27], Masahiro Ito[6,27], Hiroshi Yatsuhashi[6,27], Yoshihiko Maehara[28], Shinji Uemoto[29], Norihiro Kokudo[30], Masao Nagasaki[2,3,31], Katsushi Tokunaga[1], Minoru Nakamura[6,7,27,32,*]

[1]Department of Human Genetics, Graduate School of Medicine, the University of Tokyo, Tokyo, Japan

[2]Department of Integrative Genomics, Tohoku Medical Megabank Organization, Tohoku University, Sendai, Japan

[3]Graduate School of Medicine, Tohoku University, Sendai, Japan

[4]The Research Center for Hepatitis and Immunology, National Center for Global Health and Medicine, Ichikawa, Japan

[5]Japan Science and Technology Agency (JST), Tokyo, Japan

[6]Clinical Research Center, National Hospital Organization (NHO) Nagasaki Medical

Center, Omura, Japan

[7]Headquarters of PBC Research in NHO Study Group for Liver Disease in Japan (NHOSLJ), Clinical Research Center, NHO Nagasaki Medical Center, Omura, Japan

[8]Department of Medicine, Teikyo University School of Medicine, Tokyo, Japan,

[9]Department of Gastroenterology and Rheumatic Diseases, Fukushima Medical University of Medicine, Fukushima, Japan

[10]Department of Gastroenterology and Hepatology, Tokyo Jikei University School of Medicine, Tokyo, Japan

[11]Department of Gastroenterology and Metabology, Ehime University Graduate School of Medicine, Matsuyama, Japan),

[12]Department of Gastroenterology, Kanazawa University Graduate School of Medicine, Kanazawa, Japan

[13]Division of Gastroenterology, Department of Medicine, Kurume University School of Medicine, Kurume, Japan

[14]Department of Medicine and Gastroenterology, Tokyo Women's Medical University, Tokyo, Japan

[15]Department of Medicine, Division of Gastroenterology and Hepatology, Shinshu University School of Medicine, Matsumoto, Japan

[16]Department of Gastroenterology and Hepatology, Nagasaki University Graduate School of Biomedical Sciences, Nagasaki, Japan

[17]Department of Gastroenterology and Hepatology, Okayama University Graduate School of Medicine, Dentistry and Pharmaceutical Sciences, Okayama, Japan

[18]Division of Gastroenterology and Hepatology, Niigata University Graduate School of Medical and Dental Sciences, Niigata, Japan

[19]Faculty of Medicine, Oita University, Oita, Japan

[20]Department of Gastroenterology and Medicine, Fukuoka, University School of Medicine, Fukuoka, Japan

[21]The Third Department of Internal Medicine, School of Medicine, University of Occupational and Environmental Health, Kitakyushu, Japan

[22]Department of Medicine and Clinical Oncology, Graduate School of Medicine, Chiba University, Chiba, Japan

[23]Department of Gastroenterology, Yamagata University Faculty of Medicine, Yamagata, Japan

[24]Division of Gastroenterology and Hepatology, Department of Internal Medicine, Keio Graduate School of Medicine, Tokyo, Japan

[25]Department of Medical Technology, Kagawa Prefectural University of Health Sciences, Kagawa, Japan

[26]Department of Medicine and Biosystemic Science, Kyushu University Graduate School of Medical Sciences, Fukuoka, Japan

[27]Department of Hepatology, Nagasaki University Graduate School of Biomedical Sciences, Omura, Japan

[28]Department of Surgery and Science, Kyushu University Graduate School of Medical Sciences, Fukuoka, Japan

[29]Division of Hepato-Biliary-Pancreatic and Transplant Surgery, Department of Surgery, Graduate School of Medicine, Kyoto University, Kyoto, Japan

[30]National Center for Global Health and Medicine, Tokyo, Japan

[31]Graduate School of Information Sciences, Tohoku University, Sendai, Japan

[32]Headquaters of PBC-GWAS study group in Japan, Clinical Research Center, NHO Nagasaki Medical Center, Omura, Japan

[33]Deceased

*Correspondence should be addressed to:

Minoru Nakamura, M.D., Ph.D.

Clinical Research Center, National Hospital Organization (NHO) Nagasaki Medical Center, Department of Hepatology, Nagasaki University Graduate School of Biomedical Sciences

2-1001-1 Kubara, Omura, Nagasaki 856-8562, Japan

Tel: +81-(0)957-52-3121; Fax: +81-(0)957-53-6675

E-mail: nakamuram@nagasaki-mc.com

†These authors contributed equally

Table of contents

Supplementary Tables ……………………………………………………………7

Supplementary Figures……………………………………………………………10

Supplementary Tables

Supplementary Table 1. Demographics of PBC cases and controls used for association analysis.

	PBC cases (n=668)	Controls (n=480)
number of cases: male/female	572/90	375/104
age (years): range	25-87	20-82
median	58	34
mean ± SD	58.5 ± 11.4	37.0 ± 11.74
AMA positive (%)	86.80%	-

Supplementary Table 2. Oligo-nucleotide probes for the EMSA

Gene	SNP	Allele	Sequence (5' -> 3')
TIMMDC1	rs2293370	C	GAGAAAGAAAAGCCA**C**AGTGGTGATGCCAAA
		T	GAGAAAGAAAAGCCA**T**AGTGGTGATGCCAAA
ARHGAP31	rs56008261	C	TGAAAAGTCAAAAAA**C**AACAAAACAAAACAA
		T	TGAAAAGTCAAAAAA**T**AACAAAACAAAACAA

Supplementary Table 3. Primers for production of Luciferase assay constructs.

Gene	SNP	Primer Name	Sequence (5' -> 3')
TIMMDC1	rs2293370	rs2293370-F	GGTACCATTAGGTTGTGGGGATGGAG
		rs2293370-R	GAGCTCTGTTACAGCTGAGCCTTGAC

Supplementary Figures

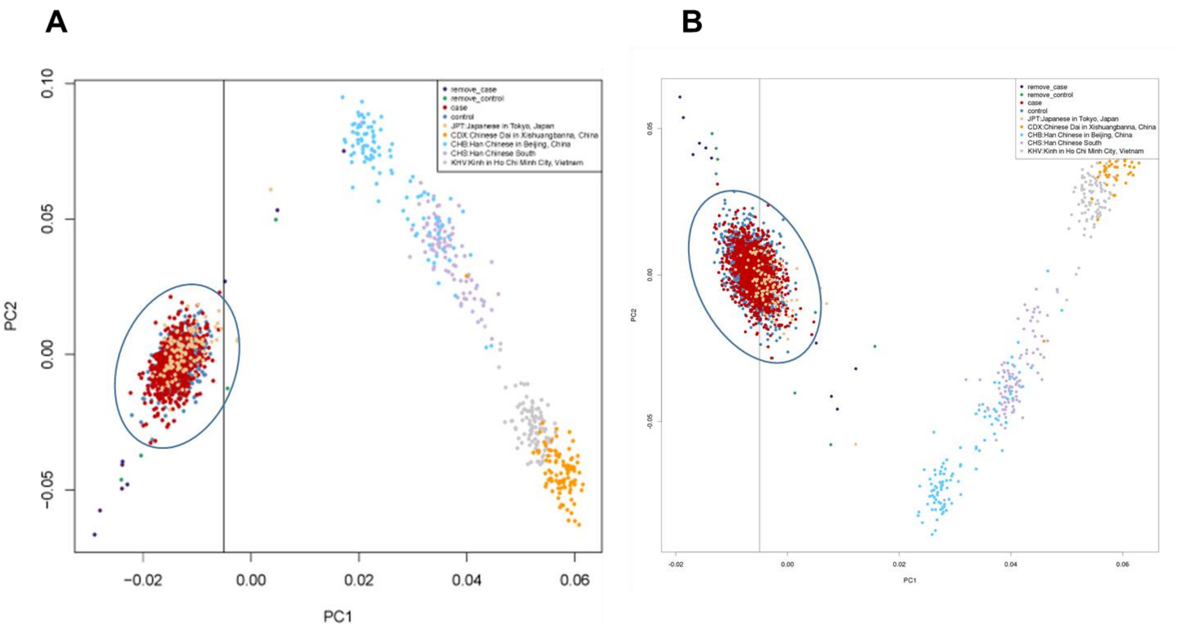

Supplementary Figure 1. Principal component analysis of samples from the GWAS stage and East Asian samples of the International 1000 Genome Project. (A) The GWAS stage from the Japonica platform. (B) The GWAS stage from the ASI platform. Individual data points are colored by ethnic origin or are our samples (see legend). The blue circle indicates the Japanese group.

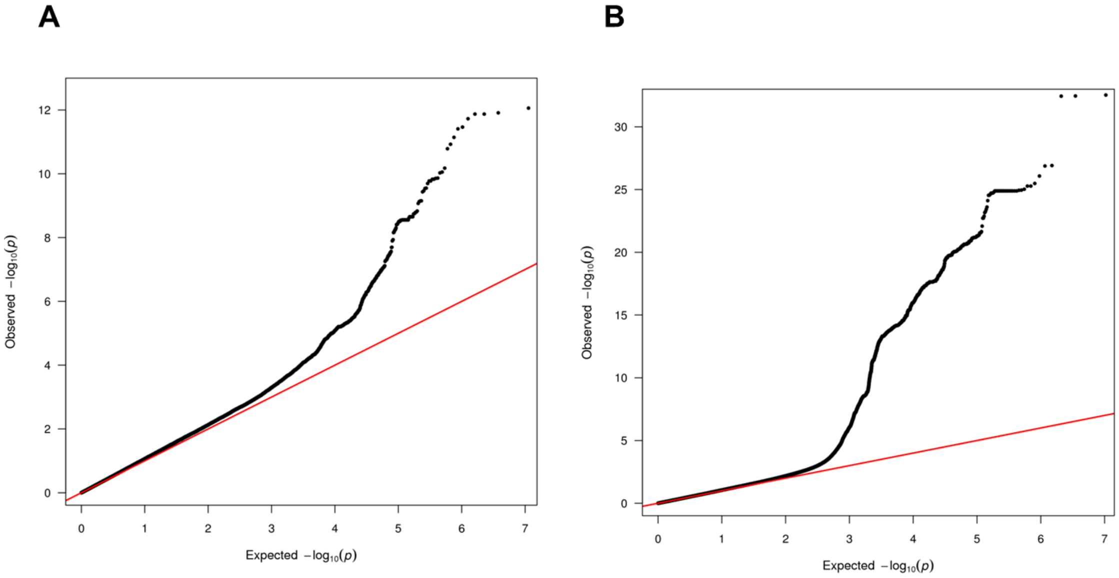

Supplementary Figure 2. Quantile–quantile plot for P-values of each SNP calculated from allele-based logistic tests for GWAS. (A) The GWAS stage from the Japonica platform. The inflation factor λ was estimated to be 1.061. (B) The GWAS stage from the ASI platform. The inflation factor λ was estimated to be 1.097.

ASI array (Kawashima *et al.* 2017)

	Genotype calling	QC fail	relatives	PCA	after exclusion
case	1392	-10	-7	-10	1365
control	1505	-8	-8	-7	1476
total	2897	-18	-15	-17	2841

Japonica array (present study)

	Genotype calling	QC fail	relatives	PCA	after exclusion
case	668	-22	-7	-9	630
control	480	-12	-6	-4	458
total	1148	-34	-13	-13	1088

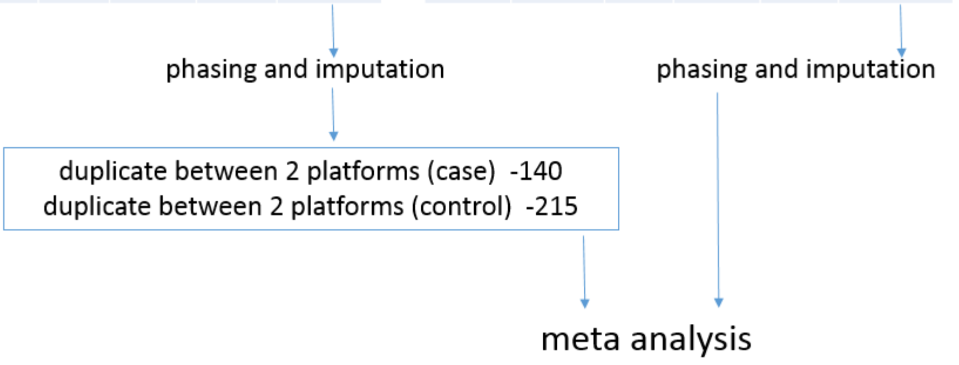

Supplementary Figure 3. Meta-analysis workflow. We performed genotype calling, phasing and imputation for each SNP array separately (ASI platform and Japonica platform). After genotype calling, individuals with a low call rate, cryptic relatives, and PCA outliers were excluded. Before meta-analysis, duplicated individuals in the two platforms were excluded from samples in the ASI platform.

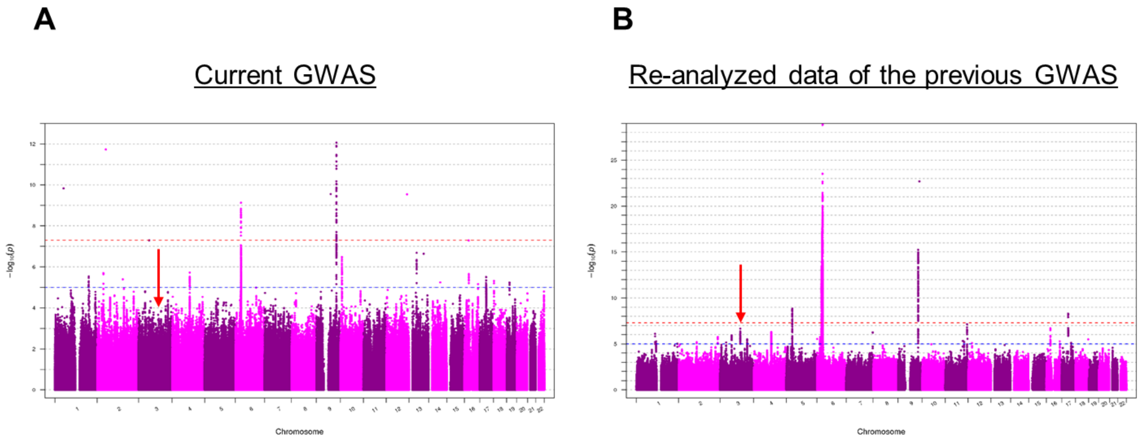

Supplementary Figure 4. Genome-wide Manhattan plot of the GWAS before meta-analysis. (A) The GWAS stage from the Japonica platform. P-values were calculated for each SNP from data for 2,897 samples (1,392 PBC cases and 1,505 healthy controls). (B) The GWAS stage from the ASI platform. P-values were calculated for each SNP from data for 1,148 samples (668 PBC cases and 480 healthy controls).

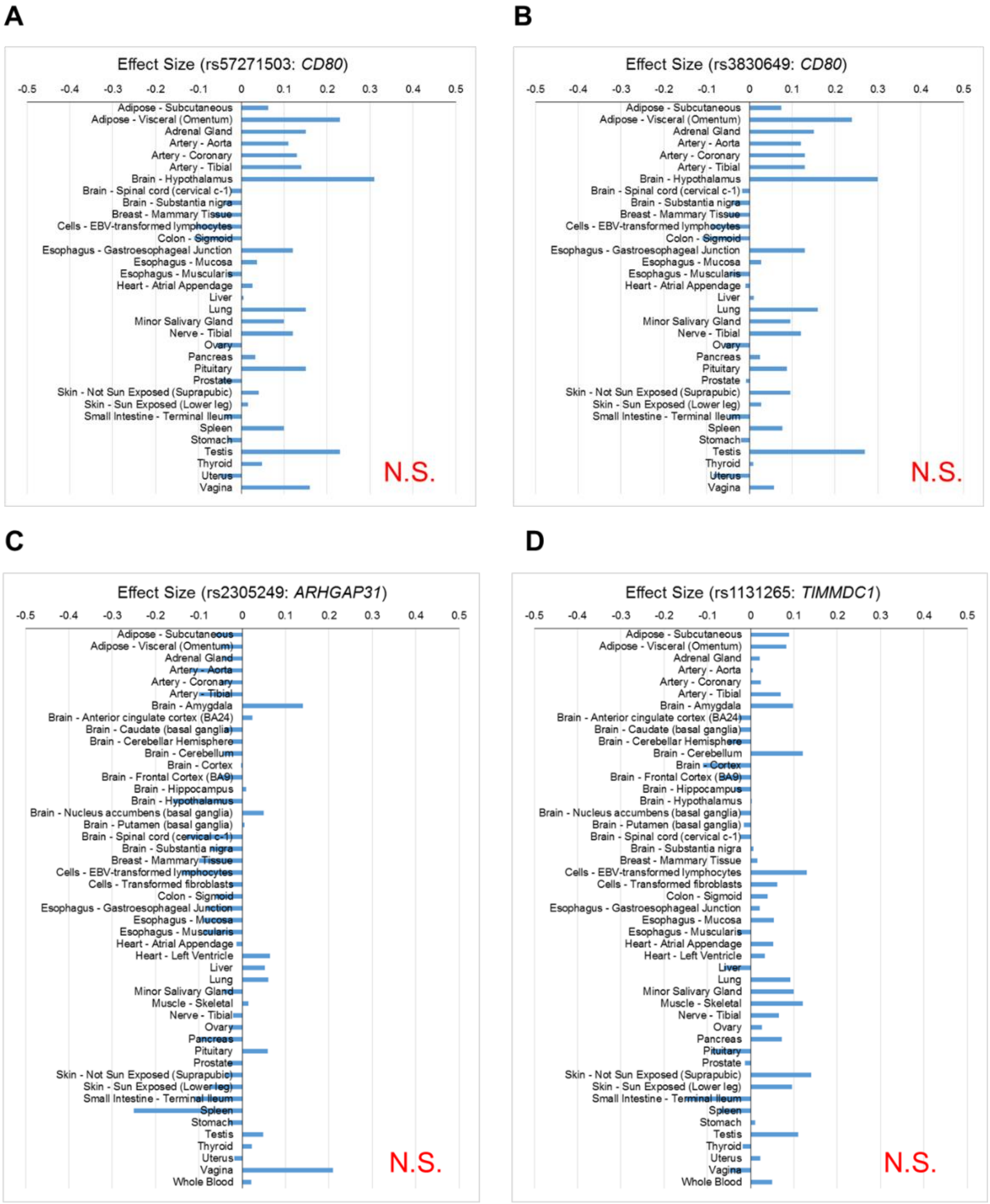
A
Effect Size (rs57271503: CD80)
-0.5 -0.4 -0.3 -0.2 -0.1 0 0.1 0.2 0.3 0.4 0.5
Adipose - Subcutaneous
Adipose - Visceral (Omentum)
Adrenal Gland
Artery - Aorta
Artery - Coronary
Artery - Tibial
Brain - Hypothalamus
Brain - Spinal cord (cervical c-1)
Brain - Substantia nigra
Breast - Mammary Tissue
Cells - EBV-transformed lymphocytes
Colon - Sigmoid
Esophagus - Gastroesophageal Junction
Esophagus - Mucosa
Esophagus - Muscularis
Heart - Atrial Appendage
Liver
Lung
Minor Salivary Gland
Nerve - Tibial
Ovary
Pancreas
Pituitary
Prostate
Skin - Not Sun Exposed (Suprapubic)
Skin - Sun Exposed (Lower leg)
Small Intestine - Terminal Ileum
Spleen
Stomach
Testis
Thyroid
Uterus
Vagina
N.S.
B
Effect Size (rs3830649: CD80)
-0.5 -0.4 -0.3 -0.2 -0.1 0 0.1 0.2 0.3 0.4 0.5
Adipose - Subcutaneous
Adipose - Visceral (Omentum)
Adrenal Gland
Artery - Aorta
Artery - Coronary
Artery - Tibial
Brain - Hypothalamus
Brain - Spinal cord (cervical c-1)
Brain - Substantia nigra
Breast - Mammary Tissue
Cells - EBV-transformed lymphocytes
Colon - Sigmoid
Esophagus - Gastroesophageal Junction
Esophagus - Mucosa
Esophagus - Muscularis
Heart - Atrial Appendage
Liver
Lung
Minor Salivary Gland
Nerve - Tibial
Ovary
Pancreas
Pituitary
Prostate
Skin - Not Sun Exposed (Suprapubic)
Skin - Sun Exposed (Lower leg)
Small Intestine - Terminal Ileum
Spleen
Stomach
Testis
Thyroid
Uterus
Vagina
N.S.
C
Effect Size (rs2305249: ARHGAP31)
-0.5 -0.4 -0.3 -0.2 -0.1 0 0.1 0.2 0.3 0.4 0.5
Adipose - Subcutaneous
Adipose - Visceral (Omentum)
Adrenal Gland
Artery - Aorta
Artery - Coronary
Artery - Tibial
Brain - Amygdala
Brain - Anterior cingulate cortex (BA24)
Brain - Caudate (basal ganglia)
Brain - Cerebellar Hemisphere
Brain - Cerebellum
Brain - Cortex
Brain - Frontal Cortex (BA9)
Brain - Hippocampus
Brain - Hypothalamus
Brain - Nucleus accumbens (basal ganglia)
Brain - Putamen (basal ganglia)
Brain - Spinal cord (cervical c-1)
Brain - Substantia nigra
Breast - Mammary Tissue
Cells - EBV-transformed lymphocytes
Cells - Transformed fibroblasts
Colon - Sigmoid
Esophagus - Gastroesophageal Junction
Esophagus - Mucosa
Esophagus - Muscularis
Heart - Atrial Appendage
Heart - Left Ventricle
Liver
Lung
Minor Salivary Gland
Muscle - Skeletal
Nerve - Tibial
Ovary
Pancreas
Pituitary
Prostate
Skin - Not Sun Exposed (Suprapubic)
Skin - Sun Exposed (Lower leg)
Small Intestine - Terminal Ileum
Spleen
Stomach
Testis
Thyroid
Uterus
Vagina
Whole Blood
N.S.
D
Effect Size (rs1131265: TIMMDC1)
-0.5 -0.4 -0.3 -0.2 -0.1 0 0.1 0.2 0.3 0.4 0.5
Adipose - Subcutaneous
Adipose - Visceral (Omentum)
Adrenal Gland
Artery - Aorta
Artery - Coronary
Artery - Tibial
Brain - Amygdala
Brain - Anterior cingulate cortex (BA24)
Brain - Caudate (basal ganglia)
Brain - Cerebellar Hemisphere
Brain - Cerebellum
Brain - Cortex
Brain - Frontal Cortex (BA9)
Brain - Hippocampus
Brain - Hypothalamus
Brain - Nucleus accumbens (basal ganglia)
Brain - Putamen (basal ganglia)
Brain - Spinal cord (cervical c-1)
Brain - Substantia nigra
Breast - Mammary Tissue
Cells - EBV-transformed lymphocytes
Cells - Transformed fibroblasts
Colon - Sigmoid
Esophagus - Gastroesophageal Junction
Esophagus - Mucosa
Esophagus - Muscularis
Heart - Atrial Appendage
Heart - Left Ventricle
Liver
Lung
Minor Salivary Gland
Muscle - Skeletal
Nerve - Tibial
Ovary
Pancreas
Pituitary
Prostate
Skin - Not Sun Exposed (Suprapubic)
Skin - Sun Exposed (Lower leg)
Small Intestine - Terminal Ileum
Spleen
Stomach
Testis
Thyroid
Uterus
Vagina
Whole Blood
N.S.

E

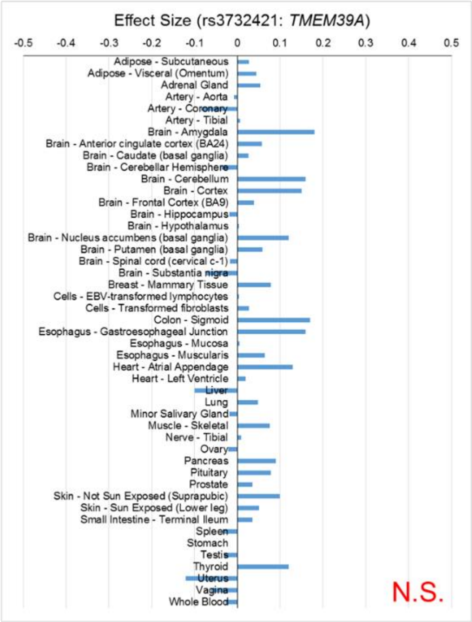

Supplementary Figure 5. SNPs located in the 3'-untranslated region (UTR) and synonymous substitutions were not related to endogenous gene expression by e-QTL analysis. Comparison between genotypes of rs57271503 and the endogenous expression of *CD80* (**A**), rs3830649 and *CD80* **(B)**, rs2305249 and *ARHGAP31* **(C)**, rs1131265 and *TIMMDC1* **(D)**, and rs3732421 and *TMEM39A* **(E)**, in all tissues registered in the GTEX database. Statistical significance levels before Bonferroni multiple comparison correction were: *P* = 0.0015 (**A** and **B**), and *P* = 0.0011 (**C** - **E**).

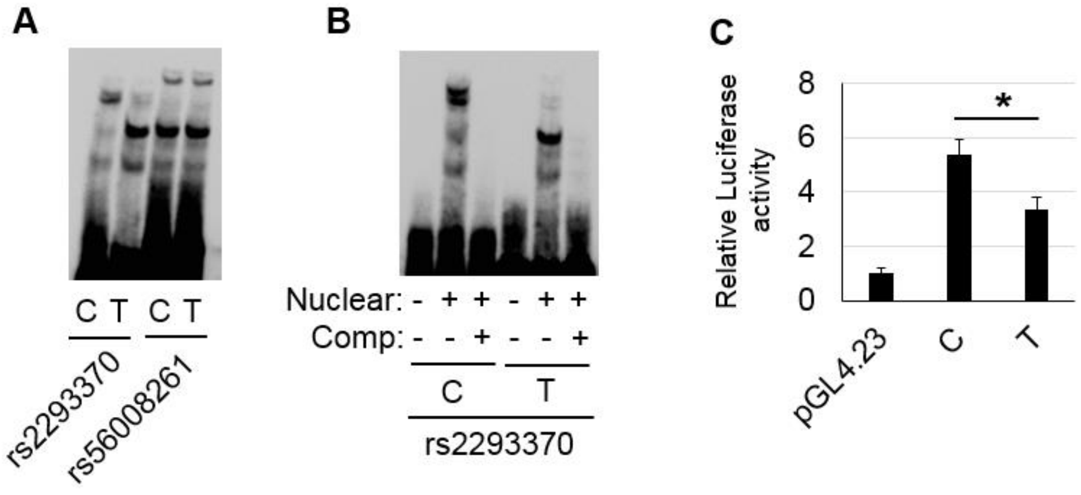

Supplementary Figure 6. ***In vitro*** **functional analysis of each candidate variation in chromosome 3q13.33 using Jurkat cells. (A)** EMSA of each candidate primary variation using biotin-labeled probes corresponding to the major and minor alleles, and nuclear extracts of Jurkat cells. rs2293370 was the only SNP to show a difference in mobility shift between the two alleles. **(B)** Competitor assay, using Jurkat nuclear extracts and a 200× concentration of unlabeled probe corresponding to either the C (i.e., PBC susceptibility) or T alleles of rs2293370. **(C)** Transcription was measured by cellular luciferase activity 24 h after transfection of the Jurkat cells. The luciferase activities of cells transfected with the PBC susceptibility allele (C allele) of rs2293370 were higher than those transfected with the T allele. Three independent experiments with triplicate measurements were performed for each assay, and data represent mean ± SD; * $P < 0.05$ (Student's *t*-test).

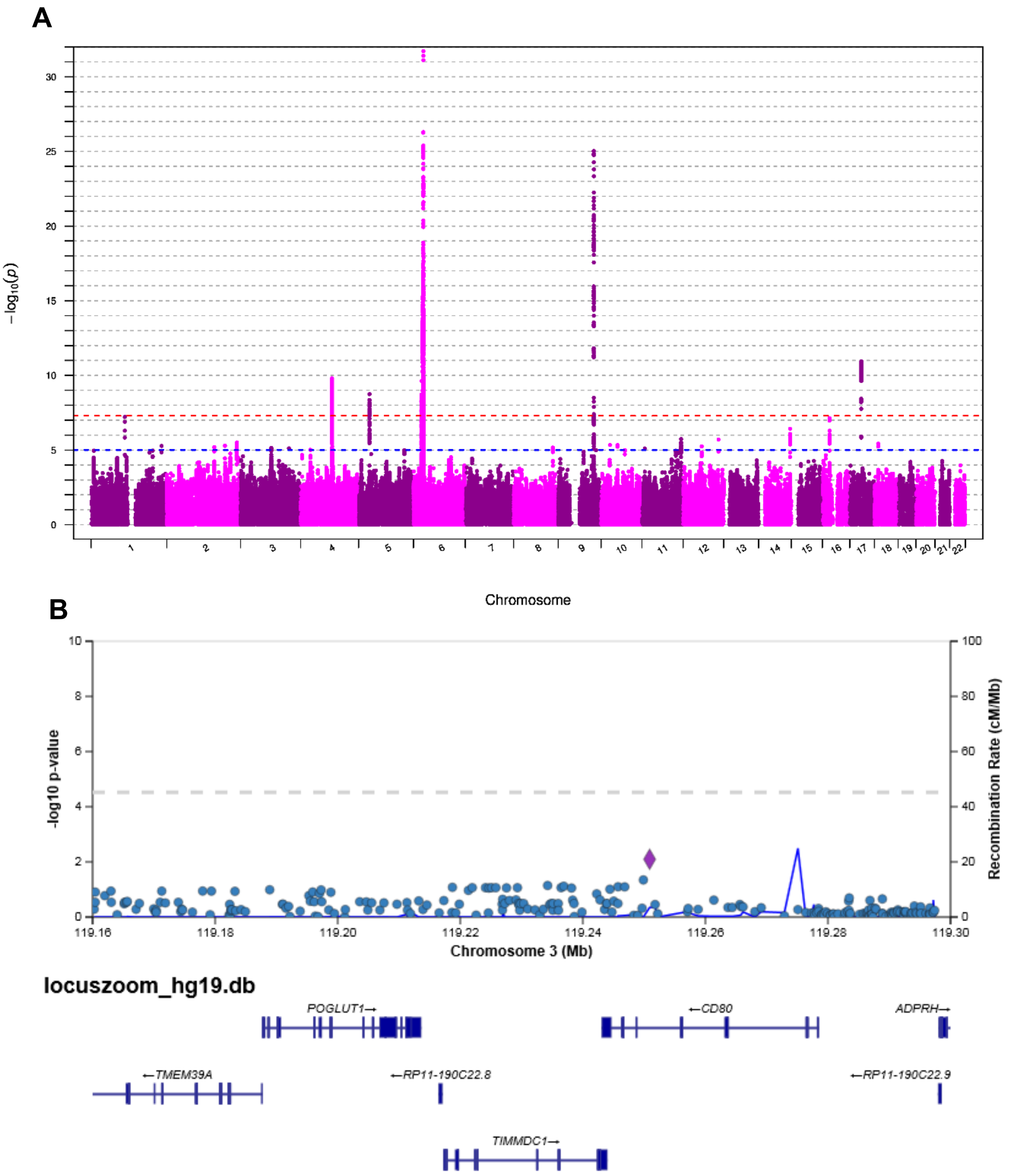

Supplementary Figure 7. Conditional logistic regression analysis of rs2293370. (A) Genome-wide Manhattan plot of the GWAS meta-analysis conditioned on rs2293370. Negative log 10 P-values of qualified SNPs plotted

against their genomic positions. The genome-wide significance line (red) is shown at 7.30 (-log10 P [5e-8]). The genome-wide suggestive line (blue) is shown at 5 (-log10 P [1e-5]). **(B)** Regional plot of association results and recombination rates for the region surrounding POGLUT1 (chromosome 3: 119,160,000-119,300,300). Each dot represents the P-value of each SNP after meta-analysis. The purple diamond represents the SNP with the minimum P-value in the region. Genetic recombination rates are shown with a blue line.

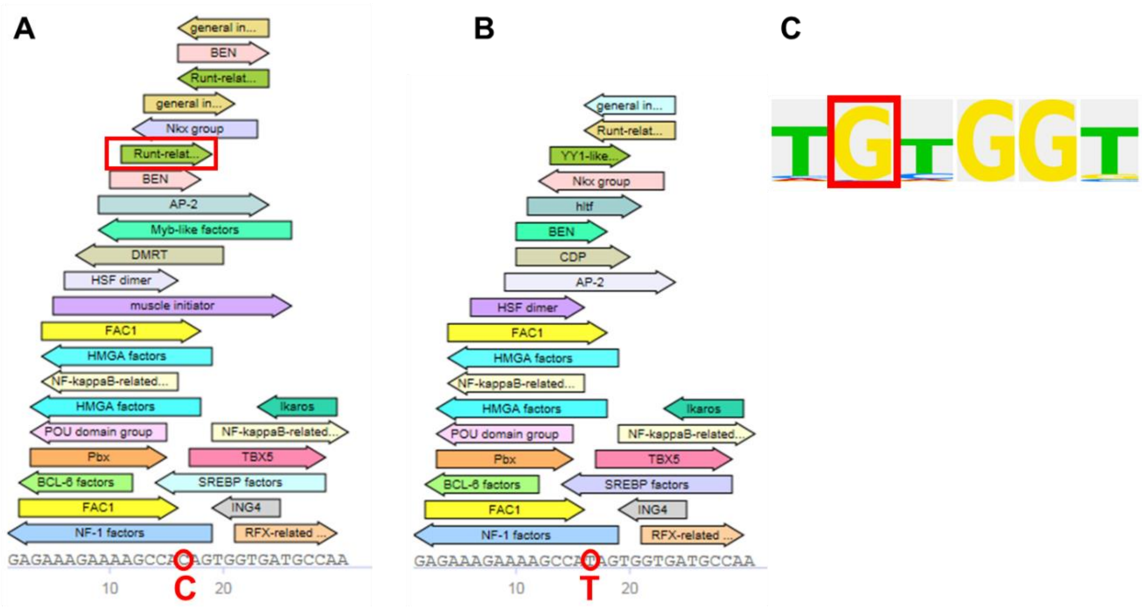

Supplementary Figure 8. Prediction of transcription factor binding using the TRANSFAC database. (A and **B)** The C allele of rs2293370 produces a Runt-related factor binding site (**A**), however, this sequence is disrupted in the T allele (**B**). **(C)** The binding motif of RUNX1, with the position of rs2293370 shown as a rectangle.

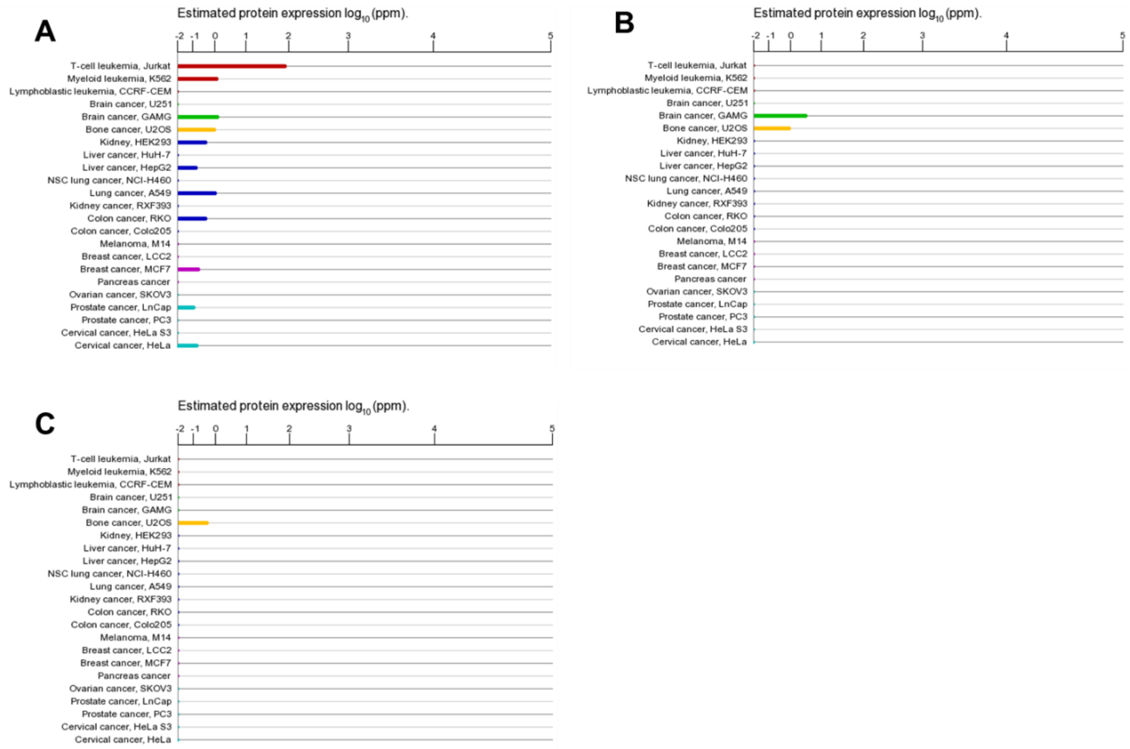

Supplementary Figure 9. Expression levels of Runt-related factors in cancer cell lines. Both Jurkat and HepG2 cells showed abundant expression of *RUNX1* (**A**), but not of *RUNX2* (**B**) or *RUNX3* (**C**). Data were obtained using GeneCards from the Weizmann Institute of Science (http://www.genecards.org/).

A

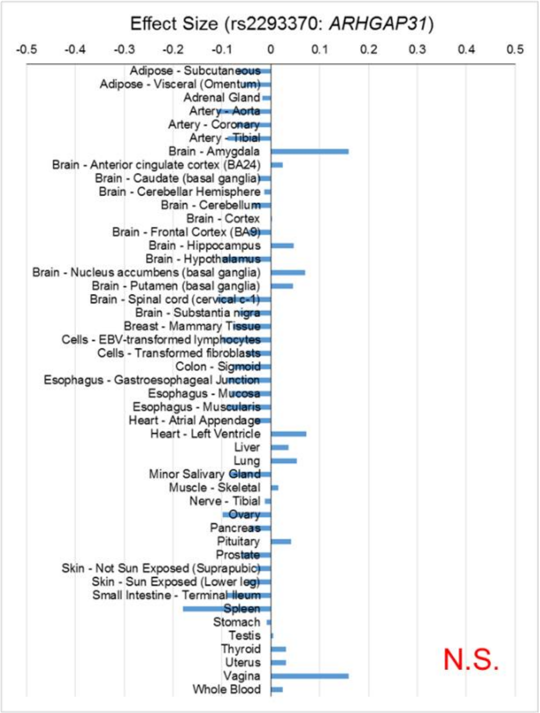

Effect Size (rs2293370: *ARHGAP31*)
-0.5 -0.4 -0.3 -0.2 -0.1 0 0.1 0.2 0.3 0.4 0.5
Adipose - Subcutaneous
Adipose - Visceral (Omentum)
Adrenal Gland
Artery - Aorta
Artery - Coronary
Artery - Tibial
Brain - Amygdala
Brain - Anterior cingulate cortex (BA24)
Brain - Caudate (basal ganglia)
Brain - Cerebellar Hemisphere
Brain - Cerebellum
Brain - Cortex
Brain - Frontal Cortex (BA9)
Brain - Hippocampus
Brain - Hypothalamus
Brain - Nucleus accumbens (basal ganglia)
Brain - Putamen (basal ganglia)
Brain - Spinal cord (cervical c-1)
Brain - Substantia nigra
Breast - Mammary Tissue
Cells - EBV-transformed lymphocytes
Cells - Transformed fibroblasts
Colon - Sigmoid
Esophagus - Gastroesophageal Junction
Esophagus - Mucosa
Esophagus - Muscularis
Heart - Atrial Appendage
Heart - Left Ventricle
Liver
Lung
Minor Salivary Gland
Muscle - Skeletal
Nerve - Tibial
Ovary
Pancreas
Pituitary
Prostate
Skin - Not Sun Exposed (Suprapubic)
Skin - Sun Exposed (Lower leg)
Small Intestine - Terminal Ileum
Spleen
Stomach
Testis
Thyroid
Uterus
Vagina
Whole Blood
N.S.

B

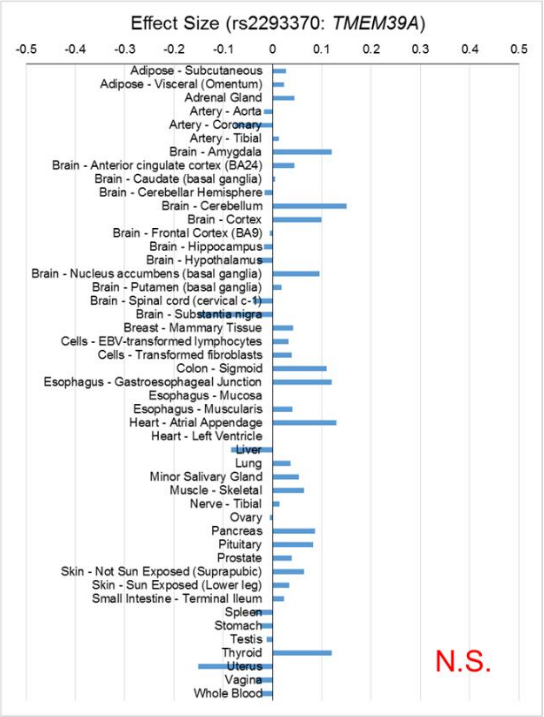

Effect Size (rs2293370: *TMEM39A*)
-0.5 -0.4 -0.3 -0.2 -0.1 0 0.1 0.2 0.3 0.4 0.5
Adipose - Subcutaneous
Adipose - Visceral (Omentum)
Adrenal Gland
Artery - Aorta
Artery - Coronary
Artery - Tibial
Brain - Amygdala
Brain - Anterior cingulate cortex (BA24)
Brain - Caudate (basal ganglia)
Brain - Cerebellar Hemisphere
Brain - Cerebellum
Brain - Cortex
Brain - Frontal Cortex (BA9)
Brain - Hippocampus
Brain - Hypothalamus
Brain - Nucleus accumbens (basal ganglia)
Brain - Putamen (basal ganglia)
Brain - Spinal cord (cervical c-1)
Brain - Substantia nigra
Breast - Mammary Tissue
Cells - EBV-transformed lymphocytes
Cells - Transformed fibroblasts
Colon - Sigmoid
Esophagus - Gastroesophageal Junction
Esophagus - Mucosa
Esophagus - Muscularis
Heart - Atrial Appendage
Heart - Left Ventricle
Liver
Lung
Minor Salivary Gland
Muscle - Skeletal
Nerve - Tibial
Ovary
Pancreas
Pituitary
Prostate
Skin - Not Sun Exposed (Suprapubic)
Skin - Sun Exposed (Lower leg)
Small Intestine - Terminal Ileum
Spleen
Stomach
Testis
Thyroid
Uterus
Vagina
Whole Blood
N.S.

C

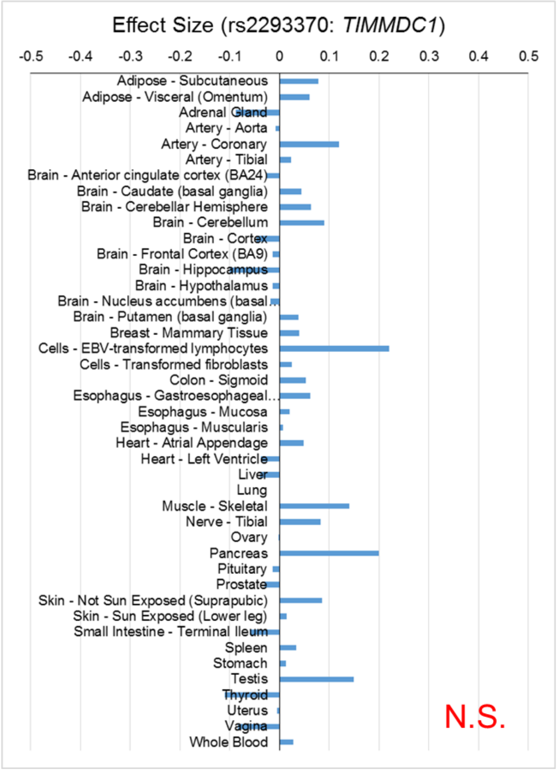

Effect Size (rs2293370: *TIMMDC1*)
-0.5 -0.4 -0.3 -0.2 -0.1 0 0.1 0.2 0.3 0.4 0.5
Adipose - Subcutaneous
Adipose - Visceral (Omentum)
Adrenal Gland
Artery - Aorta
Artery - Coronary
Artery - Tibial
Brain - Anterior cingulate cortex (BA24)
Brain - Caudate (basal ganglia)
Brain - Cerebellar Hemisphere
Brain - Cerebellum
Brain - Cortex
Brain - Frontal Cortex (BA9)
Brain - Hippocampus
Brain - Hypothalamus
Brain - Nucleus accumbens (basal…
Brain - Putamen (basal ganglia)
Breast - Mammary Tissue
Cells - EBV-transformed lymphocytes
Cells - Transformed fibroblasts
Colon - Sigmoid
Esophagus - Gastroesophageal…
Esophagus - Mucosa
Esophagus - Muscularis
Heart - Atrial Appendage
Heart - Left Ventricle
Liver
Lung
Muscle - Skeletal
Nerve - Tibial
Ovary
Pancreas
Pituitary
Prostate
Skin - Not Sun Exposed (Suprapubic)
Skin - Sun Exposed (Lower leg)
Small Intestine - Terminal Ileum
Spleen
Stomach
Testis
Thyroid
Uterus
Vagina
Whole Blood
N.S.

D

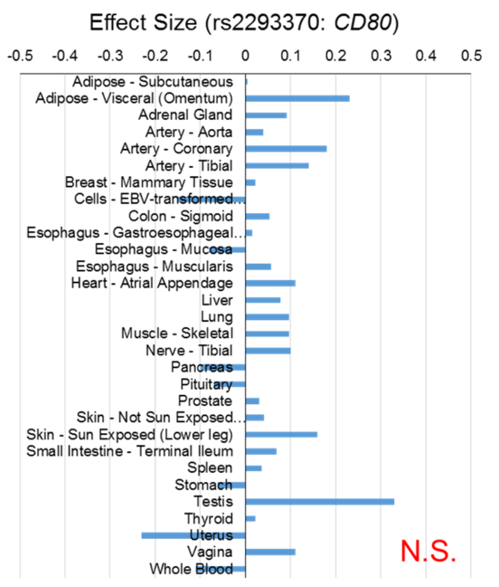

Effect Size (rs2293370: *CD80*)
-0.5 -0.4 -0.3 -0.2 -0.1 0 0.1 0.2 0.3 0.4 0.5
Adipose - Subcutaneous
Adipose - Visceral (Omentum)
Adrenal Gland
Artery - Aorta
Artery - Coronary
Artery - Tibial
Breast - Mammary Tissue
Cells - EBV-transformed…
Colon - Sigmoid
Esophagus - Gastroesophageal…
Esophagus - Mucosa
Esophagus - Muscularis
Heart - Atrial Appendage
Liver
Lung
Muscle - Skeletal
Nerve - Tibial
Pancreas
Pituitary
Prostate
Skin - Not Sun Exposed…
Skin - Sun Exposed (Lower leg)
Small Intestine - Terminal Ileum
Spleen
Stomach
Testis
Thyroid
Uterus
Vagina
Whole Blood
N.S.

Supplementary Figure 10. rs2293370 was not related to the endogenous gene expression of *ARHGAP31*, *TMEM39A*, *TIMMDC1*, and *CD80* by e-QTL analysis. Comparison between genotypes of rs2293370 and the endogenous expression of *ARHGAP31* (**A**), *TMEM39A* (**B**), *TIMMDC1* (**C**), and *CD80* (**D**), in all tissues registered in the GTEX database. The effect sizes of the rs2293370 minor allele (T allele; disease protective) in every organ are shown. Statistical significance levels before Bonferroni multiple comparison correction were: P = 0.0011 (**A** - **B**), and P = 0.00115 (**D**).

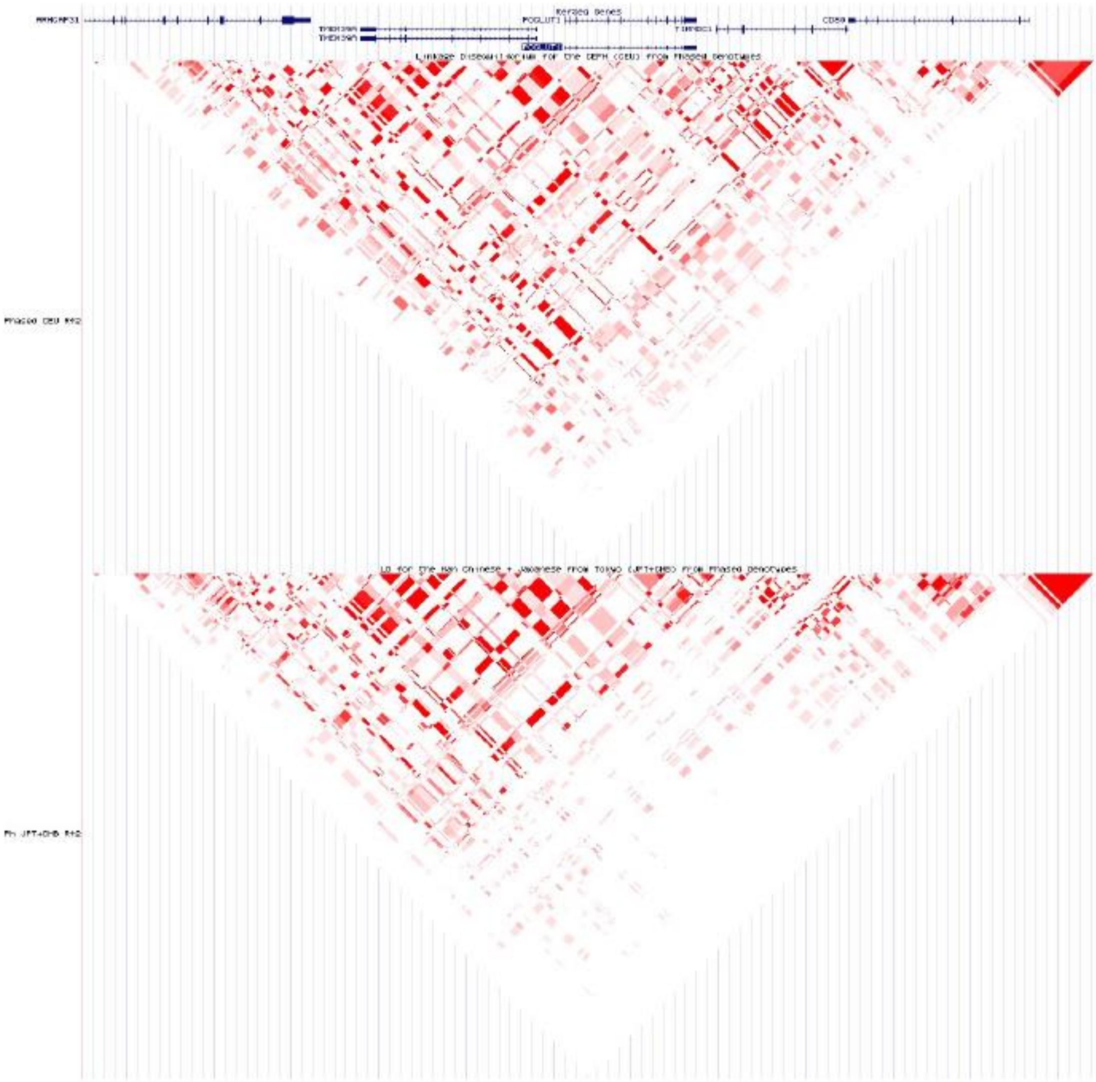

Supplementary Figure 11. LD plot of chromosome 3q13.33 in 1000 genomes European and Asian. The r^2 value are indicated by color density.
